# A Potential Role for Aminoacylation in Primordial RNA Copying Chemistry

**DOI:** 10.1021/acs.biochem.0c00943

**Published:** 2021-02-01

**Authors:** Aleksandar Radakovic, Tom H. Wright, Victor S. Lelyveld, Jack W. Szostak

**Affiliations:** † Howard Hughes Medical Institute, Department of Molecular Biology, and Center for Computational and Integrative Biology, Massachusetts General Hospital, 185 Cambridge Street, Boston, Massachusetts 02114, United States; ‡ Department of Genetics, Harvard Medical School, Boston, Massachusetts 02115, United States

## Abstract

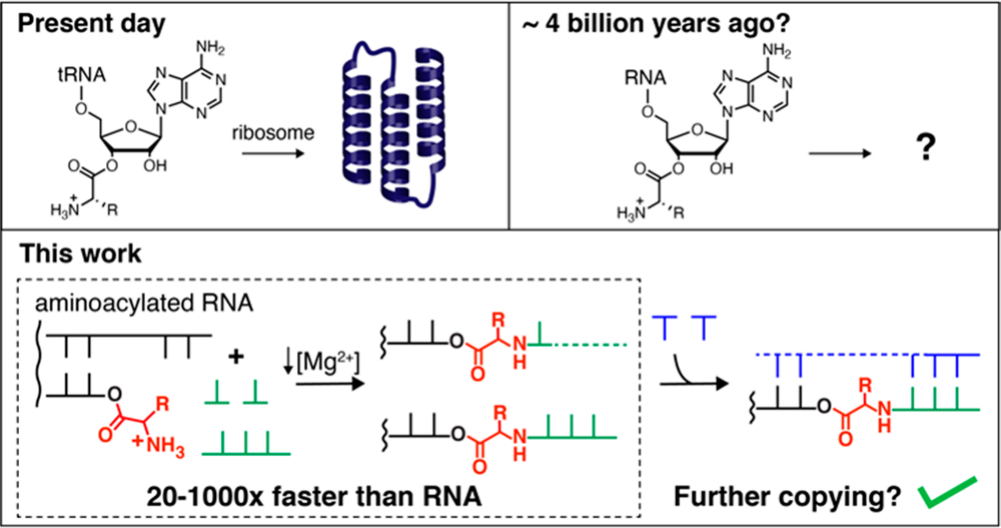

Aminoacylated tRNAs are the substrates for ribosomal protein synthesis in all branches of life, implying an ancient origin for aminoacylation chemistry. In the 1970s, Orgel and colleagues reported potentially prebiotic routes to aminoacylated nucleotides and their RNA-templated condensation to form amino acid-bridged dinucleotides. However, it is unclear whether such reactions would have aided or impeded non-enzymatic RNA replication. Determining whether aminoacylated RNAs could have been advantageous in evolution prior to the emergence of protein synthesis remains a key challenge. We therefore tested the ability of aminoacylated RNA to participate in both templated primer extension and ligation reactions. We find that at low magnesium concentrations that favor fatty acid-based protocells, these reactions proceed orders of magnitude more rapidly than when initiated from the *cis*-diol of unmodified RNA. We further demonstrate that amino acid-bridged RNAs can act as templates in a subsequent round of copying. Our results suggest that aminoacylation facilitated non-enzymatic RNA replication, thus outlining a potentially primordial functional link between aminoacylation chemistry and RNA replication.

In extant biochemistry, the aminoacylation of RNA generates activated tRNA substrates for protein biosynthesis. Explaining how RNA could be aminoacylated without enzymes, and how such aminoacylation might have benefitted early protocells, may help to explain how the RNA World underwent the transition to protein-centric biology. The covalent linkage of amino acids to RNA would have unavoidably affected RNA replication. If these effects were beneficial, then efficient ribozyme catalyzed aminoacylation could have evolved. Once in place, ribozyme catalysis of aminoacylation could in turn have led to other uses for covalently attached amino acids, such as peptide formation.

Nucleotides and RNA strands can be aminoacylated at the 2′(3′)-hydroxyl groups by reaction with amino acid imidazolides^1^ (Figure 1A), which in turn can be formed from the imidazole-catalyzed reaction of a free amino acid with a nucleotide activated as a 5′-phosphorimidazolide.^2^ However, the latter reaction competes with the rapid conversion of the aminoacyl adenylate intermediate into an *N*-carboxyanhydride (NCA) in the presence of CO_2_.^3,4^ NCAs are inefficient reagents for the direct aminoacylation of ribonucleotides.^5^ High-yielding aminoacylation pathways employing NCAs^6^ or in situ activation chemistry^7^ are known, but they require a 3′-phosphate moiety and are generally limited to N-blocked amino acids. Therefore, we still do not have a high-yielding and prebiotically plausible means of chemically aminoacylating RNA strands terminating in a vicinal diol. However, even inefficient chemistry could have had significant effects on RNA replication and assembly processes.

**Figure 1:**
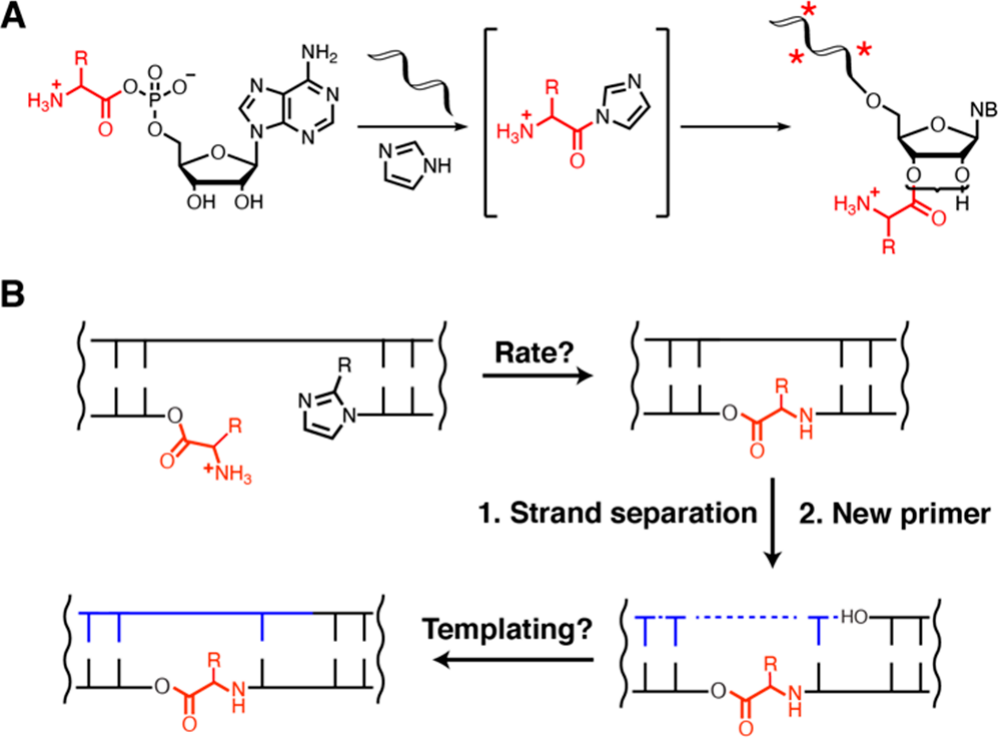
Aminoacylation of RNA and integration of covalently attached amino acids with RNA copying. (A) Aminoacylation chemistry. The reaction of imidazole with aminoacyl adenylate anhydrides generates amino acid imidazolides, which in turn serve as amino acid donors for covalent modification of RNA strands with amino acids (asterisks denote internal 2′-acylated OHs). (B) Non-enzymatic primer extension or ligation initiated from a 2′(3′)-aminoacyl-terminated RNA strand generates amino acid-bridged RNA, which can act as a template for future rounds of copying.

We were curious about whether the additional chemical functionality imparted by amino acids could assist the non-enzymatic, chemical copying of RNA strands. In 1974, Shim and Orgel reported that 2′(3′)-aminoacylated nucleotides can react with nucleotide 5′-phosphorimidazolides in a template-directed process to form phosphoramidate-linked products via transamidation.^8^ On a poly(U) template, 2′(3′)-glycyl adenosine reacted with 5′-imidazole-activated AMP to afford a dinucleotide species that was bridged by the glycine residue.^8^ Formation of such phosphoramidate-linked amino acid RNA “copolymers” dramatically increases the stability of both the aminoacyl ester and phosphoramidate bonds, suggesting a mechanism for enhanced covalent capture of amino acids by early RNA.^9^ In parallel, the Orgel laboratory^10,11^ and later our own group^12−14^ demonstrated that nucleotides with either 2′- or 3′-amino groups exhibit a large increase in the rate and yield of non-enzymatic polymerization due to the greater nucleophilicity of the amine substituent relative to that of a hydroxyl group. These results have been extended to ligation reactions by the Krishnamurthy group^15^ and our own recent assembly of an active ribozyme via a series of 3′N–5′P ligation reactions.^16,17^ While the α-amino group of aminoacylated RNA should be more nucleophilic than the hydroxyl group of RNA, it was unclear whether the different location of the α-amino group or the increased steric bulk in the reaction center would diminish the rate of primer extension or ligation reactions. Indeed, replacing the 3′-hydroxyl group with a hydroxymethyl group completely abolished reactivity.^18^ Together, these results inspired us to test whether the reaction of the free amino group of 2′(3′)-aminoacylated RNA with phosphorimidazolide-activated nucleotides would result in enhanced rates of RNA copying, and whether such amino acid-bridged oligonucleotides could act as templates for cycles of replication (Figure 1B).

Here, we report that terminal 2′(3′)-aminoacylation of RNA can indeed enhance both template-directed primer extension and ligation reactions with imidazole-activated downstream species by >2 orders of magnitude. We also show that RNA oligomers containing a single amino acid “bridge” can act as templates for subsequent rounds of RNA template copying. Taken together, our findings reveal a potential pathway by which non-enzymatic RNA aminoacylation could have facilitated non-enzymatic RNA replication. The subsequent evolution of a more efficient ribozyme-catalyzed aminoacylating activity could thus have been directly advantageous for early protocells, while at the same time generating the aminoacylated RNA substrates required for the later evolution of ribozyme-mediated peptide synthesis.

## Materials and Methods

### General Information

All reagents were purchased from Sigma-Aldrich (St. Louis, MO) unless specified otherwise. TurboDNase was purchased from Thermo Scientific (Waltham, MA). Flexizyme “dFx” and the corresponding mutant M2 were prepared as described elsewhere.^19^ Polymerase chain reaction was performed with Hot Start Taq 2X Master Mix, and in vitro transcription with the HiScribe T7 Quick High Yield RNA Synthesis Kit from New England Biolabs (Ipswich, MA). EDTA is used as an abbreviation for Na_2_EDTA (pH 8.0).

### Oligonucleotide Synthesis

Oligonucleotides were either purchased from Integrated DNA Technologies (Coralville, IA) or synthesized in house on an Expedite 8909 solid-phase oligo synthesizer. Phosphoramidites and reagents for the Expedite synthesizer were purchased from either Glen Research (Sterling, VA) or Chemgenes (Wilmington, MA). Cleavage of synthesized oligonucleotides from the solid support was performed using 1 mL of AMA (1:1 mixture of 28% aqueous ammonium hydroxide and 40% aqueous methylamine) for 30 min at room temperature, while deprotection was performed in the same solution for 20 min at 65 °C. Deprotected oligos were lyophilized, resuspended in 100 μL of DMSO and 125 μL of TEA·3HF, and heated at 65 °C for 2.5 h to remove TBDMS from 2′-hydroxyls. Following this deprotection, oligos were purified by preparative 20% polyacrylamide gel electrophoresis (19:1 with 7 M urea), desalted using Waters (Milford, MA) Sep-Pak C18 cartridges, and characterized by high-resolution mass spectrometry on an Agilent 6230 TOF mass spectrometer.

### Oligonucleotide and Nucleotide Activation

Oligonucleotides phosphorylated on the 5′-OH were activated with 2-methylimidazole or 2-aminoimidazole as previously reported^20^ with the following modifications. Gel-purified products of 1 μmol solid-phase synthesis were dissolved in 100 μL of DMSO; 0.05 mmol of triethylamine (TEA), 0.02 mmol of triphenylphosphine (TPP), 0.04 mmol of 2-methylimidazole (or 2-aminoimidazole), and 0.02 mmol of 2,2′-dipyridyldisulfide (DPDS) were added to the reaction mixture, and the reaction mixture was incubated on a rotator for 5 h at room temperature. After 5 h, all of the reagents mentioned above were added in listed quantities again and the reaction mixture was allowed to rotate for an additional 12 h at room temperature. The reaction mixture was precipitated with 100 μL of saturated NaClO_4_ in acetone and 1 mL of acetone for 30 min on dry ice. The pellet was washed twice with 1 mL of a 1:1 acetone/diethyl ether mixture. The products were resolved and purified by HPLC on an Agilent ZORBAX analytical column (Eclipse Plus C18, 250 mm × 4.6 mm, 5 μm particle size, P.N. 959990-902), at a flow rate of 1 mL/min. The following gradient was used: (A) aqueous 20 mM triethylammonium bicarbonate (pH 8.0) and (B) acetonitrile, from 7% to 12% B over 12 min.

2-Aminoimidazolium Cytidine Dinucleotide (C*C). First, 0.46 mmol of CMP (free acid) was dissolved in 4 mL of DMSO, and 2.9 mmol of TEA, 3.8 mmol of TPP, and 0.22 mmol of 2-aminoimidazole (HCl salt) were added to the CMP solution. The resulting suspension was sonicated and heated briefly until all of the reagents had completely dissolved. Four millimoles of DPDS was added to the solution to start the reaction, and the reaction mixture was stirred for 15 min at room temperature. The reaction mixture was then precipitated by adding 0.5 mL of saturated NaClO_4_ in acetone and 60 mL of a 1:1 acetone/diethyl ether mixture, washed twice with a 1:1 acetone/diethyl ether mixture, and purified by C_18_ reverse-phase chromatography at a flow rate of 40 mL/min. The following gradient was used: (A) aqueous 2 mM triethylammonium bicarbonate (pH 8.0) and (B) acetonitrile, from 0% to 10% B over 10 min.

2-Aminoimidazolium Guanosine-Uridine Dinucleotide (G*U). For OAt-GMP synthesis, 0.275 mmol of GMP (free acid) was dissolved in 18 mL of water, followed by 1.47 mmol of HOAt and 1.5 mmol of TEA. The solution was then lyophilized. The resulting powder was dissolved in 10 mL of DMSO, followed by the addition of 3.6 mmol of TEA, 2.75 mmol of TPP, and 2.75 mmol of DPDS. The reaction mixture was stirred at room temperature for 30 min, precipitated by adding 0.5 mL of saturated NaClO_4_ in acetone and 60 mL of a 1:1 acetone/diethyl ether mixture, washed twice with a 1:1 acetone/diethyl ether mixture, and purified by C_18_ reverse-phase chromatography at a flow rate of 40 mL/min. The folowing gradient was used: (A) aqueous 2 mM triethylammonium bicarbonate (pH 8.0) and (B) acetonitrile, from 0% to 15% B over 10 min. For 2-AI-UMP synthesis, UMP was activated as C*C, except 1.38 mmol of 2-aminoimidazole was added (3 equiv). Purified OAt-GMP and 2-AI-UMP were then mixed in 4 mL of water for 1 h. G*U was purified by preparative HPLC on an Agilent preparative column (Eclipse XDB C18, 250 mm × 21.2 mm, 7 μm particle size, P.N. 977250-402), at a flow rate of 15 mL/min. The following gradient was used: (A) aqueous 2 mM triethylammonium bicarbonate (pH 8.0) and (B) acetonitrile, from 2% to 8% B over 20 column volumes.

### Amino Acid Substrate Synthesis

Amino acid-DBE substrates (3,5-dinitrobenzyl esters of amino acids) were synthesized as reported previously^19^ with the following modification. *N*-Boc-protected amino acid DBE-esters were deprotected in 2 mL of neat TFA for 10 min, followed by washing with 10 mL of 3× diethyl ether. Products were obtained as TFA salts. TFA salts were dissolved in 100% DMSO to a final concentration of 25 mM and used in reactions directly. ^1^H NMR spectra were recorded using a 400 MHz NMR spectrometer (Varian INOVA) operating at 400 MHz. Low-resolution mass spectrometry was performed by directly injecting 10 μL of a 2 mg/mL solution in 1:1 acetonitrile/water mixture on an Esquire 6000 mass spectrometer (Bruker Daltonics). High-resolution mass spectrometry was performed by injecting 500 pmol of material dissolved in water on an Agilent 1200 HPLC instrument coupled to an Agilent 6230 TOF mass spectrometer.

### Flexizyme-Catalyzed Aminoacylation of Oligonucleotides (Figure S1)

Aminoacylation reactions were performed as reported previously^19^ with the following modifications. A typical 10 μL reaction mixture contained 50 mM Na-HEPES (pH 8.0), 10 mM MgCl_2_, 10 μM fluorescein-labeled primer, 5 mM aa-DBE (final DMSO concentration of 20%), and 10 μM dFx Flexizyme. The reaction mixture was incubated on ice for 12–16 h. One microliter of the reaction mixture was quenched with 9 μL of quench buffer [10 mM EDTA, 100 mM NaOAc (pH 5.0), 150 mM HCl, and 70% (v/v) formamide] and loaded into a 20% polyacrylamide gel [19:1 with 7 M urea and 0.1 M NaOAc (pH 5.0)] in a cold room (4 °C). The gel was run for 2 h at 300 V and visualized on a Typhoon 9410 imager. A typical aminoacylation reaction yielded 30–60% product, measured by band densities in ImageQuant TL software.

### Amino Acid-Bridged Oligonucleotide Synthesis (Figure S2)

A primer with the amino acid bridge before the 3′-terminal nucleotide (**1**) was synthesized as described previously^21^ with the following modifications. Oligonucleotide aminoacylation was performed at a 1 mL scale and then split into two followed by the addition of the FX_T2 hybrid template and FX_S2 “sandwich” (Table S1) to final concentrations of 2.5 μM each. Na-HEPES (pH 8.0) and EDTA were added to final concentrations of 200 and 50 mM, respectively. The solution was allowed to warm to room temperature for 2 min, after which the reaction was started by the addition of the C*C dinucleotide to a final concentration of 13.5 mM. The reaction was allowed to proceed for 10 min while the mixture was being rotated at room temperature. The reaction mixture was then concentrated using Amicon Ultra-4 mL 3K centrifugal filters, and the buffer was exchanged twice with nuclease-free water. The reaction mixtures were combined and further concentrated to 50 μL with Amicon Ultra-0.5 mL 3K centrifugal filters; 375 μL of nuclease-free water, 50 μL of 10× TurboDNase buffer, and 50 units of 2 units/μL TurboDNase were added. TurboDNase digestion was allowed to proceed for 15 min at 37 °C. The digested reaction mixture was then concentrated using Amicon Ultra-0.5 mL 3K centrifugal filters, diluted with 5 mM EDTA in 95% (v/v) formamide, and purified by preparative 20% polyacrylamide gel electrophoresis (19:1 with 7 M urea) at 4 °C. The desired gel band was cut out, crushed, and extracted with 1 mL of 50 mM NaOAc (pH 5.5)/50 mM EDTA buffer on a rotator at 4 °C for 16 h. The extracted product was concentrated using Amicon Ultra-0.5 mL 3K centrifugal filters, and the buffer was exchanged thrice with nuclease-free water to yield 80–90% pure **1**.

A template with an internal amino acid bridge (**2**) was synthesized by performing a ligation reaction with the following modifications. Oligonucleotide aminoacylation was performed at a 1 mL scale and then split into two followed by the addition of the NP DNA T template (Table S1) to a final concentration of 2.5 μM. Na-HEPES (pH 8.0) and EDTA were added to final concentrations of 200 and 50 mM, respectively. The solution was allowed to warm to room temperature for 2 min, after which the reaction was started by the addition of 2-methylimidazole-activated Ligator1 (Table S1) to a final concentration of 10 μM. The reaction was allowed to proceed for 1 h while the mixture was being rotated at room temperature. The reaction mixture was concentrated with Amicon Ultra-4 mL 3K centrifugal filters, and the buffer was exchanged twice with nuclease-free water. The reaction mixture was further concentrated to 50 μL using Amicon Ultra-0.5 mL 3K centrifugal filters. DNA digestion and purification were performed exactly as described for **1**. After gel extraction and buffer exchange, the 90% pure **2** was precipitated with 0.1 volume of 5 M NH_4_OAc and 3 volumes of isopropanol.

### Primer Extension Reactions

#### With the C*C Dinucleotide (Figures 2–4, Figure S5, and Figure S6)

The RNA template and the downstream RNA oligonucleotide (“sandwich”) were added to a typical 10 μL aminoacylation reaction mixture to final concentrations of 3.75 and 2.5 μM, respectively, followed by Na-HEPES (pH 8.0) to a final concentration of 200 mM. MgCl_2_ was added to a final concentration of 50 mM for reactions that were performed at 50 mM MgCl_2_. Water was added to mixtures for reactions performed at 2.5 mM MgCl_2_. EDTA was added to mixtures for reactions performed at 0 mM MgCl_2_ to a final concentration of 25 mM. Note that because the aminoacylation reactions were performed in the presence of 10 mM MgCl_2_, they contributed 2.5 mM MgCl_2_ to the final reaction. The reaction mixtures were allowed to warm to room temperature for 2 min before the reactions were initiated by the addition of the C*C dimer to a final concentration of 20 mM. Final reaction concentrations: 2.5 μM mixture of primers, 0–50 mM MgCl_2_, 200 mM HEPES (pH 8.0), and 20 mM C*C. Reactions were performed in technical triplicates. At indicated time points, 1 μL of each reaction was quenched with 29 μL of quench buffer [final quench buffer concentrations of 50 mM EDTA, 2 μM reverse complement of the template, and 90% (v/v) formamide]. Prior to being loaded on 20% polyacrylamide gels (19:1 with 7 M urea), the quenched reaction mixtures were heated at 92 °C for 2 min to denature the duplex. Aliquots (3 μL) were loaded into gels and run at 20 W for 80 min. The gels were imaged on a Typhoon 9410 imager, and band densities quantified in ImageQuant TL software.

To independently determine the kinetics of pure RNA primer extension, primer extension was performed with the non-aminoacylated RNA primer. The non-aminoacylated primer was subjected to typical aminoacylation conditions, except dFx Flexizyme was replaced with water for those reactions. The primer extension reactions were then set up exactly as in the preceding paragraph.

The rate of hydrolysis of the aminoacylated primer was measured under primer extension conditions except that CMP was used instead of C*C. The hydrolysis reaction was quenched using the acidic quench buffer [10 mM EDTA, 100 mM NaOAc (pH 5.0), 150 mM HCl, 2 μM reverse complement of the template, and 70% (v/v) formamide], the mixture heated at 92 °C for 2 min to denature the duplex, and the reaction run on an acidic 20% polyacrylamide gel [19:1 with 7 M urea and 0.1 M NaOAc (pH 5.0)].

#### With the C*C Dinucleotide (Figure S7)

The purified product **1** and the downstream “sandwich” were annealed to the RNA template in a solution containing 3.6 μM **1**, 3.6 μM “sandwich”, 5.4 μM template, 50 mM Na-HEPES (pH 7.5), 50 mM NaCl, and 1 mM EDTA by being heated for 3 min at 70 °C and slowly cooled to 20 °C at a rate of 0.1 °C/s. The annealed solution was diluted with Na-HEPES (pH 8.0) and MgCl_2_, before the reaction was initiated by adding the C*C dinucleotide. The final reaction concentrations were 0.6 μM **1**, 200 mM Na-HEPES (pH 8.0), 50 mM MgCl_2_, and 20 mM C*C. The reaction was quenched at the indicated time points, subjected to gel electrophoresis, and quantified as described above.

#### With the G*U Dinucleotide (Figure 6 and Figure S12)

The primers and the corresponding downstream “sandwich” oligonucleotides were annealed to the template (either the glycine-linked **2** or the all-RNA template) in a solution containing 3.6 μM primer, 3.6 μM “sandwich”, 5 μM template, 50 mM Na-HEPES (pH 7.5), 50 mM NaCl, and 1 mM EDTA by being heated for 3 min at 70 °C and slowly cooled to 20 °C at a rate of 0.1 °C/s. The annealed solution was diluted with Na-HEPES (pH 8.0) and MgCl_2_, before the reaction was initiated by adding the G*U dinucleotide. The final reaction concentrations were 0.6 μM primer, 200 mM Na-HEPES (pH 8.0), 100 mM MgCl_2_, and 20 mM G*U. The reaction was quenched at the indicated time points, subjected to gel electrophoresis, and quantified as described above.

### Kinetic Analysis of Primer Extension Reactions

#### With the C*C Dinucleotide (Figures 2 and 4 and Figure S5)

For the RNA reaction, primer extension was quantified for each time point by integrating the band intensity in each gel lane. The band intensity was normalized in each lane. The remaining primer (*P*) at each time point, starting from the initial fraction of primer (*P*_0_), was plotted as −ln(*P*/*P*_0_) versus reaction time, and the observed rate constant, *k*_obs_, was estimated by the slope of a linear regression line. This *k*_obs_ corresponded to *k*_3_ in the kinetic model used to obtain the *k*_obs_(*k*_1_) of the aminoacylated primer.

For the hydrolysis reaction, hydrolysis was quantified for each time point by integrating the band intensity in each gel lane. The band intensity was normalized in each lane. The remaining primer-gly (*P*) at each time point, starting from the initial fraction of primer-gly (*P*_0_), was plotted as −ln(*P*/*P*_0_) versus reaction time, and the observed rate constant, *k*_obs_, was estimated by the slope of a linear regression line. This *k*_obs_ corresponded to *k*_2_ in the kinetic model used to obtain the *k*_obs_(*k*_1_) of the aminoacylated primer.

For the aminoacylated reaction, to obtain a rate constant for extension of the aminoacylated primer under conditions saturating for the 2-aminoimidazolium-bridged dinucleotide, we modeled the reaction with the following simplified kinetic scheme.
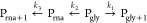
In this reaction network, P_rna_ is the native RNA primer, which can be formed by hydrolysis from P_gly_, the glycyl-terminal primer. The respective +1 species are corresponding extended products of each form of the primer. The total observable primer concentration is *P* = *P*_gly_ + *P*_rna_, and the normalized extent of initial aminoacylation of the primer is governed by the expression *P*_rna_0__ = 1 – *P*_gly_0__. Under the assumption of pseudo-first-order kinetics, the reaction can be described by the following system of differential equations.
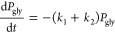
1
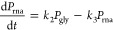
2
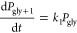
3
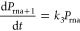
4Integrating eq 1 yields an expression for the consumption of the glycyl-terminal primer.
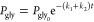
5The differential eq 3 can be integrated with eq 5, yielding an expression for the extension of the glycyl-terminal primer to form the +1 product.
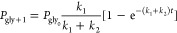
6Finally, substituting eq 5 into eq 2 and integrating gives an expression for the consumption of the native RNA primer.
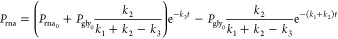
7Given independent estimates for *P*_gly_0__, *k*_2_, and *k*_3_, rate constant *k*_1_ was estimated from the normalized, integrated gel band intensity for *P* and *P*_gly+1_ by nonlinear fitting to the system of closed-form solutions (eqs 5–7). The propagated error on the estimate for *k*_1_, based on errors on the measurements for *P*_gly_0__, *k*_2_, and *k*_3_, was simulated by the Monte Carlo method.^22^

With the C*C Dinucleotide (Figure S7). Primer extension was quantified for each time point by integrating the band intensity in each gel lane. The band intensity was normalized in each lane. The remaining primer (*P*) at each time point, starting from the initial fraction of primer (*P*_0_), was plotted as −ln(*P*/*P*_0_) versus reaction time, and the observed rate constant, *k*_obs_, was estimated by the slope of a linear regression line.

With the G*U Dinucleotide (Figure 6 and Figure S12). The reaction was performed exactly as in the preceding paragraph.

### Base Hydrolysis of the +1 NP Product (Figure 3B)

A primer extension reaction performed at 2.5 mM MgCl_2_ was allowed to proceed for 40 min before being quenched with 29 μL of quench buffer [final quench concentrations of 50 mM EDTA, 2 μM reverse complement of the template, and 90% (v/v) formamide]. Two microliters of 1.5 M NaOH was added to 24 μL of the quenched reaction mixture, which increased the pH of the quenched primer extension reaction mixture to 12. The alkaline reaction mixture was incubated at room temperature for 30 s before being neutralized with 1 μL of 0.5 M HCl to pH 8. Aliquots (3 μL) were subjected to 20% polyacrylamide gel electrophoresis (19:1 with 7 M urea) and imaged as the aforementioned primer extension reactions.

### Chemical N-Acetylation of Aminoacylated Primers (Figure 3C)

Four microliters of 180 mM sulfo-NHS-acetate was added to the mixture for a typical aminoacylation reaction performed at a 40 μL scale, and the reaction mixture was incubated for 2 h at room temperature. The reaction mixture was precipitated with 0.1 volume of 5 M NH_4_OAc and 3 volumes of isopropanol on dry ice for 20 min and pelleted at 15000 rpm for 15 min at 4 °C. The pellet was washed twice with 80% ethanol and resuspended in 21 μL of nuclease-free water. Ten microliters of the precipitated reaction mixture was subjected to the primer extension procedure described in the preceding paragraphs. Because MgCl_2_ from the aminoacylation reaction was washed away during precipitation, MgCl_2_ was added to a final concentration of 2.5 mM in the final reaction. In addition, exact primer concentrations after precipitation could not be accurately determined due to the presence of dFx Flexizyme. Primer concentrations were estimated on the basis of a mock aminoacylation reaction from which dFx Flexizyme was omitted. The mock aminoacylation reaction mixture was subjected to sulfo-NHS-acetate labeling as described above and used as the “RNA” control.

### Biotin Modification of Primer Extension Reactions (Figure 3D)

Primer extension reactions were performed as described in the preceding paragraphs. At the indicated time points, 1 μL of the reaction was quenched in 14 μL of freshly prepared NHS-biotin buffer (final concentrations of 33 mM EDTA and 1 mM NHS-biotin after quenching) and incubated at room temperature for 1 h to allow for biotin labeling. At 1 h, 15 μL of quench buffer [final quench concentrations of 26 mM EDTA, 1 μM reverse complement of the template, and 46% (v/v) formamide] was added to each labeling reaction mixture. The quenched reaction mixtures were heated at 92 °C for 2 min, and 3 μL aliquots were loaded into 20% polyacrylamide gels (19:1 with 7 M urea). The gels were run at 20 W for 80 min, imaged, and quantified as the aforementioned primer extension reactions.

### Ligation Reactions (Figure 5 and Figures S8–S11)

Aminoacylation reactions that were used in subsequent ligation experiments were performed with dFx Flexizyme mutant M2 (Table S1), which recognizes the 3′-terminal ACA sequences. The RNA template was added to a typical 10 μL aminoacylation reaction mixture to a final concentration of 3.75 μM, followed by Na-HEPES (pH 8.0) to a final concentration of 200 mM. Note that because aminoacylation reactions were performed in the presence of 10 mM MgCl_2_, they contributed 2.5 mM MgCl_2_ to the final reaction. The reaction mixtures were allowed to warm to room temperature for 2 min before the reactions were initiated by the addition of the 2-methylimidazole-activated decamer [Ligator1 (Table S1)] to a final concentration of 10 μM. The final reaction concentrations were 2.5 μM mixture of primers, 2.5 mM MgCl_2_, 200 mM HEPES (pH 8.0), and 10 μM activated decamer. Reactions were performed in technical triplicates. At the indicated time points, 1 μL of each reaction was quenched with 29 μL of quench buffer [final quench buffer concentrations of 50 mM EDTA, 2 μM reverse complement of the template, and 90% (v/v) formamide]. Prior to being loaded on 20% polyacrylamide gels (19:1 with 7 M urea), the quenched reaction mixtures were heated at 92 °C for 2 min to denature the duplex. Aliquots (3 μL) were loaded into gels and run at 20 W for 80 min. The gels were imaged on a Typhoon 9410 imager, and band densities quantified in ImageQuant TL software.

To independently determine the kinetics of pure RNA ligation, ligation reactions were performed with the non-aminoacylated RNA primer. The non-aminoacylated primer was subjected to typical aminoacylation conditions, except dFx Flexizyme M2 was replaced with water for those reactions. The ligation reactions were then set up exactly as in the preceding paragraph. Note that to control for possible effects of the different amino acid-DBE esters in the RNA ligation reactions, RNA control reactions were performed in the presence of each tested amino acid-DBE ester (see Figure S8H).

Hydrolysis rates of aminoacylated primers were measured under the same ligation conditions described above, except that unactivated 10mer was used instead of the 2-methylimidazole-activated one. The hydrolysis reaction was quenched using the acidic quench buffer [10 mM EDTA, 100 mM NaOAc (pH 5.0), 150 mM HCl, 2 μM reverse complement of the template, and 70% (v/v) formamide], heated at 92 °C for 2 min to denature the duplex, and run on an acidic 20% polyacrylamide gel [19:1 with 7 M urea and 0.1 M NaOAc (pH 5.0)].

### Kinetic Analysis of Ligation Reactions

For the RNA reaction, primer extension was quantified for each time point by integrating the band intensity in each gel lane. The band intensity was normalized in each lane. The remaining primer (*P*) at each time point, starting from the initial fraction of primer (*P*_0_), was plotted as −ln(*P*/*P*_0_) versus reaction time, and the observed rate constant, *k*_obs_, was estimated by the slope of a linear regression line. This *k*_obs_ corresponds to *k*_3_ in the kinetic model used to obtain the *k*_obs_(*k*_1_) of the aminoacylated primer.

For the hydrolysis reaction, hydrolysis was quantified for each time point by integrating the band intensity in each gel lane. The band intensity was normalized in each lane. The remaining primer-gly (*P*) at each time point, starting from the initial fraction of primer-gly (*P*_0_), was plotted as −ln(*P*/*P*_0_) versus reaction time, and the observed rate constant, *k*_obs_, was estimated by the slope of a linear regression line. This *k*_obs_ corresponded to *k*_2_ in the kinetic model used to obtain the *k*_obs_(*k*_1_) of the aminoacylated primer.

The aminoacylated reaction was performed using the model described for the primer extension of aminoacylated primers with the following modifications. Because the gel band corresponding to the ligation product of aminoacylated primers could not be resolved from the gel band corresponding to the ligation product of pure RNA primers, only time points at which pure RNA primers produced <2% of the ligated product band were used to model the aminoacylated primer ligation.

### Hydrolysis Reactions (Table 1 and Figures S3 and S4)

Purified products **1** and **2** were subjected to primer extension conditions in single-stranded and double-stranded states without the addition of activated dinucleotides or decamers. Double-stranded reaction mixtures were annealed by being heated to 70 °C for 3 min and slowly cooled to 20 °C at a rate of 0.1 °C/s (single-stranded reaction mixtures were not subjected to annealing). The hydrolysis reaction conditions included 0.375 μM oligonucleotide (**1** or **2**), 200 mM Na-HEPES (pH 8.0), 2.5 or 100 mM MgCl_2_, and 22 °C (thermocycler). The reactions were stopped by the addition of quench buffer [final quench concentrations of 50 mM EDTA, 2 μM reverse complement of the template, and 90% (v/v) formamide], and the mixtures flash-frozen in liquid nitrogen. The double-stranded quenched reaction mixtures were heated for 2 min at 92 °C to denature the duplex before being loaded into the gel (the single-stranded reaction mixtures were not heated).

*Kinetic Analysis*. Hydrolysis was quantified for each time point by integrating the band intensity in each gel lane. The band intensity was normalized in each lane. The remaining amino acid-bridged oligonucleotide (*P*; either **1** or **2**) at each time point, starting from the initial fraction of the amino acid-bridged oligonucleotide (*P*_0_; either **1** or **2**), was plotted as −ln(*P*/*P*_0_) versus reaction time, and the observed rate constant, *k*_obs_, was estimated by the slope of a linear regression line. The half-lives were calculated from the *k*_obs_ values by the following equation for a first-order process: *t*_1/2_ = ln(2)/*k*_obs_.

*Characterization of the Degradation Product of **2***. The reaction was performed for 24 h in a solution that contained 0.375 μM **2**, 200 mM Na-HEPES (pH 8.0), and 2.5 mM MgCl_2_ at 22 °C. After 24 h, the reaction mixture was precipitated with 0.1 V 5 M NH_4_OAc and 3 V isopropanol, pelleted at 15000 rpm and 4 °C, washed twice with 80% ethanol, desalted using a C18 Zip-tip column, and analyzed on an Agilent 1200 HPLC instrument coupled to an Agilent 6230 TOF mass spectrometer.

## Results

**Figure 2:**
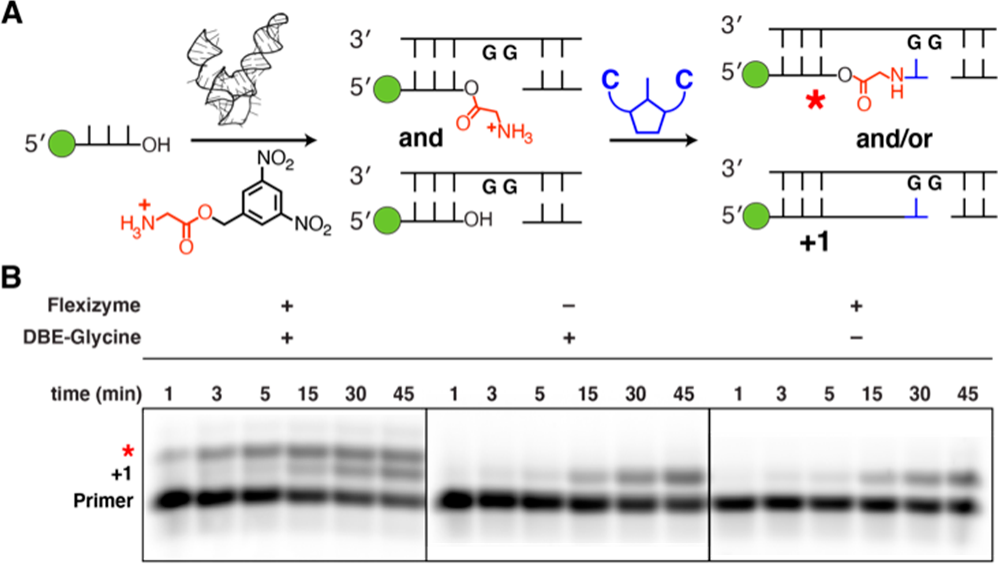
Non-enzymatic copying of RNA initiated from 2′(3′)-aminoacyl-terminated RNA. (A) Schematic of the assay used. Flexizyme acylation of a fluorescently labeled (a green circle denotes fluorescein) RNA primer results in a mixture of acylated and non-acylated strands that are used in primer extension with the 5′–5′ aminoimidazolium-bridged cytidine dinucleotide, C*C. (B) Time course of primer extension monitored using polyacrylamide gel electrophoresis. All reactions were performed at pH 8.0, 200 mM HEPES, and 2.5 mM MgCl_2_ with 20 mM C*C.

To ask whether RNA aminoacylation would interfere with or potentiate RNA copying chemistry, we first established a primer extension assay for RNA copying initiated from a 2′(3′)-aminoacylated RNA primer (Figure 2A). To aminoacylate primers we used Flexizyme, a ribozyme originally evolved by *in vitro* selection to aminoacylate any tRNA of interest with a wide variety of amino acids.^23,24^ dFx Flexizyme can aminoacylate any RNA sequence that ends in the CA-3′ sequence found in tRNAs.^24^ Although aminoacylation occurs at the 3′-hydroxyl, rapid transacylation yields a dynamic mixture of 2′- and 3′-aminoacylated regioisomers.^25^ We designed a 10-nucleotide primer terminating in CCA and tested it for aminoacylation using dFx and dinitrobenzyl-activated glycine, the dinitrobenzyl group providing the recognition element for this class of Flexizyme. Acylation yields ranging from 26% to 60% were obtained, as assessed by polyacrylamide gel electrophoresis (PAGE) under acidic conditions (Figure S1).

To investigate the ability of the 2′(3′)-glycyl oligonucleotide to act as a primer, we designed a template that provides a single binding site for a 5′–5′ aminoimidazolium-bridged cytidine dinucleotide (C*C), the reactive species in primer extension using 2-aminoimidazole-activated cytidine ribonucleotides (Figure 2B).^26^ We diluted the RNA acylation reaction mixture into primer extension buffer, added the template strand and MgCl_2_ (2.5 mM), and initiated primer extension by addition of the C*C dinucleotide (20 mM). Note that due to the short half-life of the ester linkage of 2′(3′)-glycyl RNA under primer extension conditions (Figure S5E,F), and the fact that acylation does not proceed to completion, a mixture of 2′(3′)-glycyl and native 2′,3′-hydroxyl-terminated RNA was always present in our Flexizyme-treated reaction mixtures. These two primer species are not separated by PAGE employing Tris-borate EDTA (TBE) buffer, although the products of primer extension can be resolved. Upon analysis of the reaction mixture, two new bands were observed, which we hypothesized to be due to +1 extension from either 2′(3′)-glycyl RNA or native RNA (Figure 2B). Control reactions without either Flexizyme or the glycyl dinitrobenzyl ester displayed only one product band. In addition, the inclusion of either Flexizyme or amino acid dinitrobenzyl ester did not interfere with the rate of primer extension at the concentrations employed (*vide infra*).

**Figure 3:**
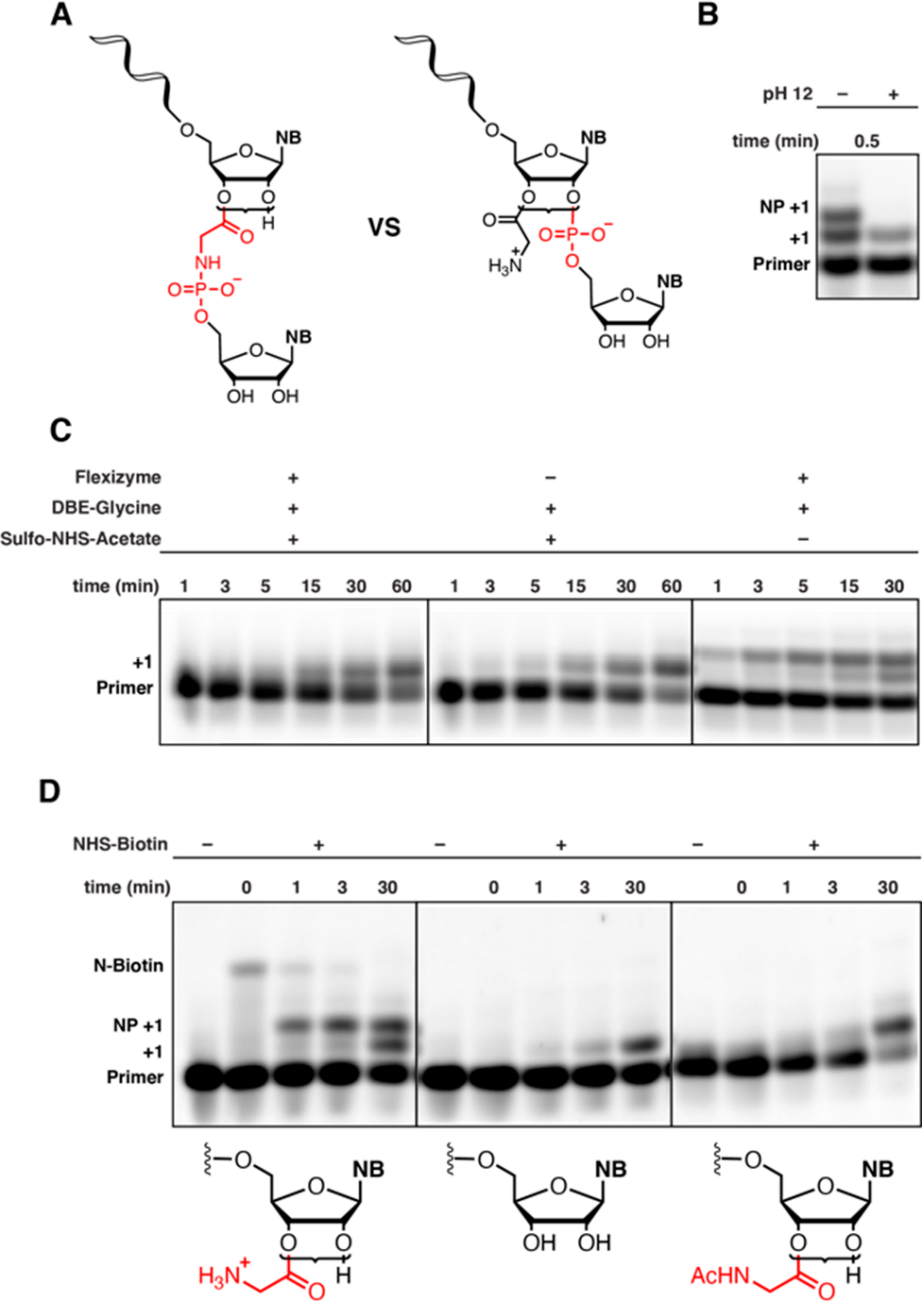
Confirmation of the presence of an amino acid “bridge”. (A) Possible reaction products of non-enzymatic copying initiated from 2′(3′)-aminoacyl-terminated RNA. Brackets indicate a dynamic mixture of 2′(3′)-aminoacylated RNA. (B) Treatment with NaOH leads to the disappearance of the novel “NP+1” band, suggesting the presence of an aminoacyl ester. (C) Chemical N-acetylation prevents the appearance of the novel “NP+1” band, suggesting that the glycyl amino group is required for formation of the novel +1 product. (D) Formation of the phosphoramidate-linked product inhibits reaction with NHS-biotin. As the primer is extended, the concentration of the glycyl amino group decreases due to N–P bond formation, leading to a reduced level of labeling with the biotinylation reagent. All reactions were performed at pH 8.0, 200 mM HEPES, and 2.5 mM MgCl_2_ with 20 mM C*C dimer.

In principle, primer extension of the aminoacylated primer by reaction with the activated 5′-phosphate of the incoming imidazolium-bridged dinucleotide could occur in either of two ways (Figure 3A). Attack of the free amino group of the 2′(3′)-glycyl RNA would result in the formation of a phosphoramidate linkage, while attack of the remaining 2′(3′)-hydroxyl of the primer would lead to the formation of a phosphodiester linkage. To first confirm the retention of an aminoacyl ester linkage in the reaction products, we subjected the reaction mixture to transient strongly basic conditions (Figure 3B). Treatment with 115 mM NaOH (pH 12) for 30 s led to the disappearance of the novel (top) +1 band, while leaving the +1 band due to extension from 2′,3′-hydroxyl-terminated RNA (bottom) unaffected, indicating the presence of a base-sensitive linkage in the top band. To test whether the free glycyl amino group was needed for extension, we performed primer extension reactions in which the acylated primer was treated with an acetylating reagent (sulfo-NHS-acetate) before the addition of the C*C imidazolium-bridged dinucleotide (Figure 3C); 2.5 mM MgCl_2_ was used in these assays to allow detection of both +1 bands at earlier time points, as the unmodified RNA primer reacts slowly under these conditions. Reaction mixtures thus treated were identical to those obtained from control reactions lacking Flexizyme, indicating that the nucleophilic glycyl amino group is required for formation of the novel +1 product. Finally, to directly prove that the free amino group is consumed during primer extension, we employed biotin labeling (Figure 3D). In this experiment, NHS-biotin was added to the primer extension reaction quench solution at each time point. If a free amino group is present, NHS-biotin will react, leading to a clear gel shift. Conversely, if no amino group is present due to N–P bond formation, no gel shift due to biotin labeling is possible. As shown in Figure 3D, as the reaction with the 2′(3′)-glycyl RNA proceeds, the extent of labeling with biotin decreases as the intensity of the +1 band increases (left panel). No labeling is observed with chemically acetylated 2′(3′)-glycyl RNA (right panel) or 2′,3′-hydroxyl-terminated RNA (middle panel). Taken together, the results from base hydrolysis and chemical labeling experiments strongly support the formation of a phosphoramidate linkage during primer extension from 2′(3′)-glycyl RNA.

To investigate the reactivity of 2′(3′)-aminoacylated, phosphoramidate-linked RNA (“amino acid-bridged RNA”), we required a means of isolating single-stranded RNA containing site-specific phosphoramidate linkages, to study primer extension, ligation, and hydrolysis. We adapted our recently reported strategy for the generation of site-specific 3′–5′ pyrophosphate linkages to provide a means to access these unusual amino acid-bridged RNAs (Figure S2).^27^ To obtain 2′(3′)-aminoacylated, phosphoramidate-linked RNA containing only a single-nucleotide extension (“terminal” amino acid-bridged RNA), we first aminoacylated an RNA primer using dFx Flexizyme. Following acylation, we performed a primer extension reaction using a DNA:RNA hybrid template in which the region to be copied is RNA but the remainder of the template is DNA. The template contains only a single binding site for the activated C*C imidazolium-bridged dinucleotide. Incubating the primer template duplex with the C*C dinucleotide leads to robust conversion of the primer to extended products in which the +1 nucleotide is connected to the terminal amino group of the 2′(3′)-aminoacylated RNA by a phosphoramidate linkage. We omitted Mg^2+^ from the primer extension reaction to discourage extension of the fraction of the RNA primer that was not acylated in the Flexizyme reaction. DNase digestion of the template then facilitated recovery of the modified primer, which we purified using preparative gel electrophoresis. To obtain RNA strands in which the amino acid linkage is followed by a longer stretch of ribonucleotides, we replaced the primer extension reaction with a ligation reaction, using a ligator RNA oligonucleotide bearing a 2-methylimidazole group activating the 5′-phosphate. In this case, the entire template was DNA so that following ligation, DNase treatment and preparative gel electrophoresis enabled purification of the modified, amino acid-linked strand.

We compared the chemical stability of amino acid-bridged RNA to that of canonical RNA, to determine whether it could, in principle, support the replication of genetic information and enable ribozyme function. Liu et al. previously measured a pH–rate profile for the breakdown of a methyl-tyrosine-linked dinucleotide as a model for the behavior of amino acid-bridged RNA co-polymers.^9^ The reported optimum stability at pH ∼6.0 reflects a balance between acid-catalyzed hydrolysis of the phosphoramidate bond and base-catalyzed hydrolysis of the aminoacyl ester linkage. The stability of the aminoacyl ester linkage was greatly enhanced upon phosphoramidate formation, potentially providing a mechanism for the stable capture of amino acids by RNA strands in a prebiotic setting.

Next, we examined the stability of longer amino acid-bridged RNAs under conditions relevant to RNA copying chemistry. We first prepared two single-stranded RNAs containing either a terminal (AGAGAAGCAA-gly-C, **1**) or an internal 2′(3′)-glycyl phosphoramidate linkage (AGAGAAGAGAGCAGACA-gly-CCCGGCAGCU, **2**), using the strategies outlined above. The 2′(3′)-glycyl, phosphoramidate-linked RNAs were then incubated at 22 °C in a pH 8.0 solution (conditions typical for primer extension reactions) at a high (100 mM) or low (2.5 mM) concentration of Mg^2+^, and in the presence or absence of a complementary strand (Table 1 and Figure S3). The maximum stability was observed for duplex products at low concentrations of Mg^2+^. Under these conditions, we observed half-lives of 53 h for terminal linkages and 72 h for internal linkages. In the presence of 100 mM Mg^2+^, conditions employed for template copying experiments (see below), the stability was decreased, although the observed half-lives on the order of hours are still sufficient to enable amino acid-linked RNAs to act as templates for further copying cycles (*vide infra*).

**Table 1:** Hydrolytic Stability of a Terminal (**1**) or Internal (**2**) 2′(3′)-Glycyl, Phosphoramidate Linkage^a^

	half-life (h^–1^)	
	ssRNA	dsRNA
terminal N–P linkage (**1**)		
100 mM MgCI_2_	10.9(3)	20.2(3)
2.5 mM MgCI_2_	24.3(4)	53(1)
internal N–P linkage (**2**)		
100 mM MgCI_2_	8.6(6)	22.8(2)
2.5 mM MgCI_2_	20.1(9)	72(2)

a All reactions were performed at pH 8.0 and 200 mM HEPES. Values are reported as the mean with the standard deviation (*N* = 3) reported at the appropriate significant digit in parentheses.

As the gel-based assay does not report on the specific linkage cleaved (ester vs phosphoramidate), we desalted samples from the cleavage of **2** after 24 h and analyzed the reaction products by LC-MS. Only products resulting from ester cleavage could be observed (Figure S4), consistent with the previously reported stability of the phosphoramidate linkage at pH >5.0.^9^

**Figure 4:**
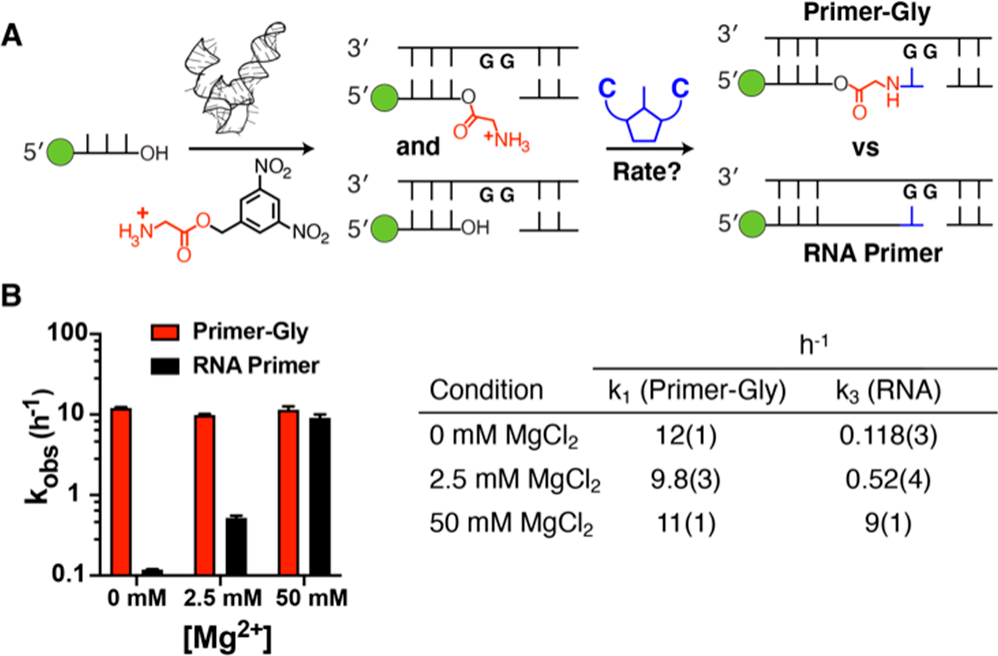
Kinetic analysis of non-enzymatic primer extension from 3′-hydroxyl- and 2′(3′)-glycyl-terminated primers. (A) Schematic representation of the assay. (B) Kinetic parameters: (left) *k*_obs_ (h^–1^) vs Mg^2+^ concentration and (right) pseudo-first-order rate constants *k*_1_, for extension of the aminoacylated primer, and *k*_3_, the native RNA primer extension rate constant, at 0, 2.5, and 50 mM MgCl_2_. Rate constants were estimated by nonlinear regression using values for *k*_2_, the aminoacyl hydrolysis rate constant, and the initial fraction of aminoacylated primer *P*_gly_0__ that were estimated from independent measurements. Plots used to obtain *k*_1_–*k*_3_ can be found in Figure S5. The uncertainty in the estimate of *k*_1_ was analyzed by the Monte Carlo method to propagate error on the estimates of independent model parameters. All reactions were performed at pH 8.0 and 200 mM HEPES with 20 mM C*C dimer. Values are reported as the mean and the standard deviation (*N* = 3) reported at the appropriate significant digit in parentheses.

The proportion of amino acid-bridged RNAs within a prebiotic population of polynucleotides would depend on the rates of amino acid activation and aminoacylation of RNA, and the competing rates of aminoacyl ester hydrolysis and phosphoramidate linkage formation. To investigate the rate of primer extension via phosphoramidate formation, we measured the rates of primer extension on a template designed to provide a single binding site for the C*C imidazolium-bridged dinucleotide (Figure 4 and Figure S5). To quantify the kinetics of glycyl-RNA primer extension, we used a simplified model of the reaction (for details, see the Supporting Information). At the start of the reaction, due to incomplete acylation by Flexizyme, both aminoacylated and non-acylated primer species are present, which cannot be resolved by gel electrophoresis. We monitored the primer extension reaction by PAGE, which can resolve the two different +1 extended reaction products, with or without a bridging amino acid residue. To follow the reaction kinetics, we modeled the hydrolysis of the aminoacylated primer to the native RNA primer as a first-order irreversible reaction with a rate constant *k*_2_. The pseudo-first-order rate constant, *k*_1_, for extension of the aminoacylated primer was estimated by nonlinear regression using independently measured values for the aminoacyl hydrolysis rate constant, *k*_2_, the native RNA primer extension rate constant, *k*_3_, and the initial fraction of aminoacylated primer *P*_gly_0__ (Figure S5).

By following the procedures outlined above, we obtained estimated rates for primer extension reactions using primers terminated in either a 2′–3′ *cis*-diol or a 2′(3′)-glycyl group (Figure 4 and Figure S5). We note that the rates we report combine possible reactions initiated from both 2′- and 3′-linked glycyl residues. At pH 8.0 and 50 mM Mg^2+^, the rate of primer extension via phosphoramidate bond formation was similar to that observed for phosphodiester bond formation (*k*_1_ = 11 h^–1^ vs 9 h^–1^ for RNA). We have previously observed that N–P bond formation using 2′- or 3′-amino groups is insensitive to Mg^2+^ concentration.^12,13^ This feature is highly desirable if genetic copying chemistry is to be integrated within fatty acid vesicles, as concentrations of free Mg^2+^ of >4 mM degrade and precipitate such membranes. We therefore performed the same primer extension reactions with and without 2.5 mM Mg^2+^. The observed rates for primer extension via phosphoramidate bond formation were insensitive to Mg^2+^, whereas phosphodiester bond formation was much slower at lower Mg^2+^ concentrations. In the absence of Mg^2+^, the rate of primer extension for the 2′(3′)-glycyl-terminated primer was 2 orders of magnitude greater than for extension from the canonical diol-terminated RNA (*k*_1_ = 12.0 h^–1^ vs 0.118 h^–1^ for RNA). These results are in accordance with the greater nucleophilicity of the amino substituent relative to the hydroxyl group and the presumed requirement for divalent metal-mediated deprotonation of the 3′-hydroxyl to afford the Mg-bound alkoxide, the most likely active species for primer extension of canonical RNA. The much greater reactivity of the glycyl-terminated RNA raised the question of whether primer extension from this modified primer is still dependent on the template. However, we found that primer extension in the presence of the template yields 76% extended product after 15 min compared to only 6% after 30 min in the absence of the template (Figure S6).

The incorporation of mismatched bases^28^ and noncanonical nucleotides^29^ can stall primer extension, presumably due to the suboptimal geometry of the reaction center. To see if an amino acid bridge would interfere with proper pairing of the terminal primer-template base pair, we quantified the rate of the reaction for a primer in which the terminal 3′-nucleotide is joined by a phosphoramidate linkage to an upstream glycine “bridge” (Figure S7). The observed rate for extension downstream of the amino acid bridge was similar to that obtained for an identical primer containing only phosphodiester linkages (10.7 h^–1^ vs 8.8 h^–1^ for RNA). Thus, the incorporation of a bridging amino acid does not significantly retard downstream primer extension steps.

In addition to the polymerization of activated nucleotides, RNA templates can also be copied by the ligation of short oligomers.^16^ This scenario is attractive as ligation requires fewer chemical reaction steps than primer extension to copy a template of a given length. However, rates of RNA ligation are much lower than for polymerization;^20^ consequently, loss of the activating group competes with ligation, leading to overall low yields. The slow rate of RNA ligation can be explained by the fact that the leaving group is simply a protonated imidazole, which lacks the highly preorganized structure of the imidazolium-bridged dinucleotide intermediate of primer extension. In fact, short oligoribonucleotides ending with 3′-amino-2′,3′-dideoxyribonucleotides show ligation rates that are orders of magnitude faster than those of all-RNA oligonucleotides.^16^ However, no potentially prebiotic route to 3′-amino nucleotides is yet known. We therefore wondered whether the more prebiotically plausible 2′(3′)-aminoacylated RNA would show similar trends in rate and yield for non-enzymatic ligation.

To compare the rates of ligation of unmodified RNA and aminoacylated RNA, we used a ligation assay similar to that developed for primer extension and the same formalism to model the kinetic system, assuming saturation of the primer-template duplex with the ligator. For ligation reactions, we were able to determine the rate of formation of the ligated product from aminoacylated RNA by collecting data at time points at which reaction with the control RNA primer was negligible. As input to our kinetic model, we measured the rates of hydrolysis and the rates of control reactions with an RNA primer in the presence of the amino acid dinitrobenzyl ester (Figure S8).

**Figure 5:**
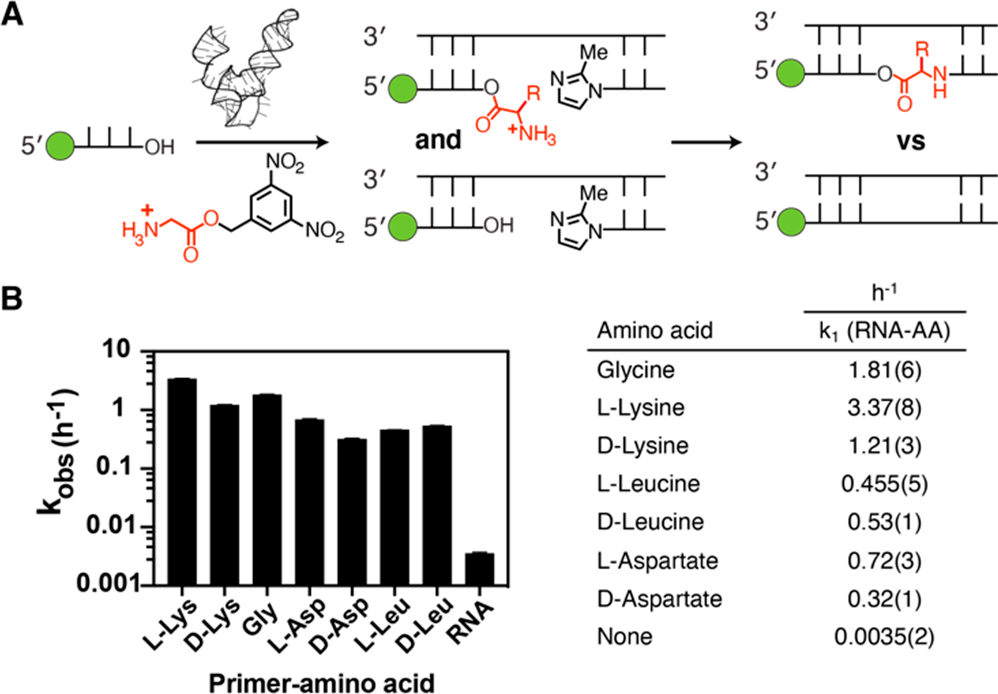
Kinetic analysis of non-enzymatic ligation from 3′-hydroxyl and 2′(3′)-aminoacyl primers. (A) Schematic representation of the assay. (B) Kinetic parameters. The *k*_obs_ values are plotted vs the primer amino acid tested. “None” refers to the unmodified RNA reaction in the presence of DBE-glycine but in the absence of Flexizyme. The pseudo-first-order rate constant, *k*_1_, for ligation of the aminoacylated primer was estimated by nonlinear regression using values for *k*_3_, the native RNA ligation rate constant, *k*_2_, the aminoacyl hydrolysis rate constant, and the initial fraction of aminoacylated primer Pgly_0_, which were measured independently. Plots used to obtain *k*_1_–*k*_3_ can be found in Figure S8. Uncertainty in the estimate of *k*_1_ was analyzed by the Monte Carlo method to propagate the error on the estimates of independent model parameters. All reactions were performed at pH 8.0, 200 mM HEPES, and 2.5 mM MgCl_2_. Values are reported as the mean with the standard deviation (*N* = 3) reported at the appropriate significant digit in parentheses.

We first tested the template-directed ligation of a 2′(3′)-glycyl primer with either a 2-methylimidazole- or 2-aminoimidazole-activated decamer (Figure S9). Although 2-aminoimidazole is a superior activating group for non-enzymatic polymerization, due to enhanced formation of the active imidazolium-bridged dinucleotide intermediate, 2-methylimidazole activation is superior for N–P ligation.^16^ This is consistent with the fact that 2-aminoimidazole is an intrinsically worse leaving group, due to its higher p*K*_a_.^30^ Indeed, in our system, the observed rate of ligation was 6-fold greater for the 2MeI-activated ligator (1.81 h^–1^ vs 0.281 h^–1^). Notably, the rate of ligation for the 2′(3′)-glycyl primer reacting with a 2-methylimidazole-activated ligator, at 2.5 mM Mg^2+^, was ∼500 times greater than for the equivalent reaction with unmodified RNA (Figure 5 and Figure S9). In the absence of the template, no ligation was observed on the time scale of the experiment (Figure S10).

Having demonstrated enhanced ligation rates with aminoacylated RNA, we were interested in determining whether the ligation rate would differ significantly across a panel of amino acids (Figure 5 and Figure S8). We tested eight amino acids that differ in charge, size, and stereochemistry. All amino acids, except for *N*^α^-acetyl-l-lysine, reacted orders of magnitude faster than RNA under the conditions of the assay (pH 8.0 and 2.5 mM Mg^2+^). l-Lys reacted at the greatest rate, which is perhaps surprising given its length and positively charged side chain. Acetylation of the lysine α-amino group blocked ligation completely, while acetylation of the ε-amino group decreased the rate 3-fold, confirming regioselectivity for reaction of the α-amine versus the ε-amine (Figures S8 and S11). Lysine and aspartate both displayed a preference for reaction of the l-enantiomer, although leucine displayed no such preference. Overall, it is notable that amino acids with different properties could be incorporated into RNA via ligation. For example, the carboxyl and amino side chains introduced by aspartate and lysine, respectively, have no parallel in native RNA. Such integration of novel functionality may allow for the expansion of the catalytic repertoire of ribozymes assembled by non-enzymatic ligation.

**Figure 6:**
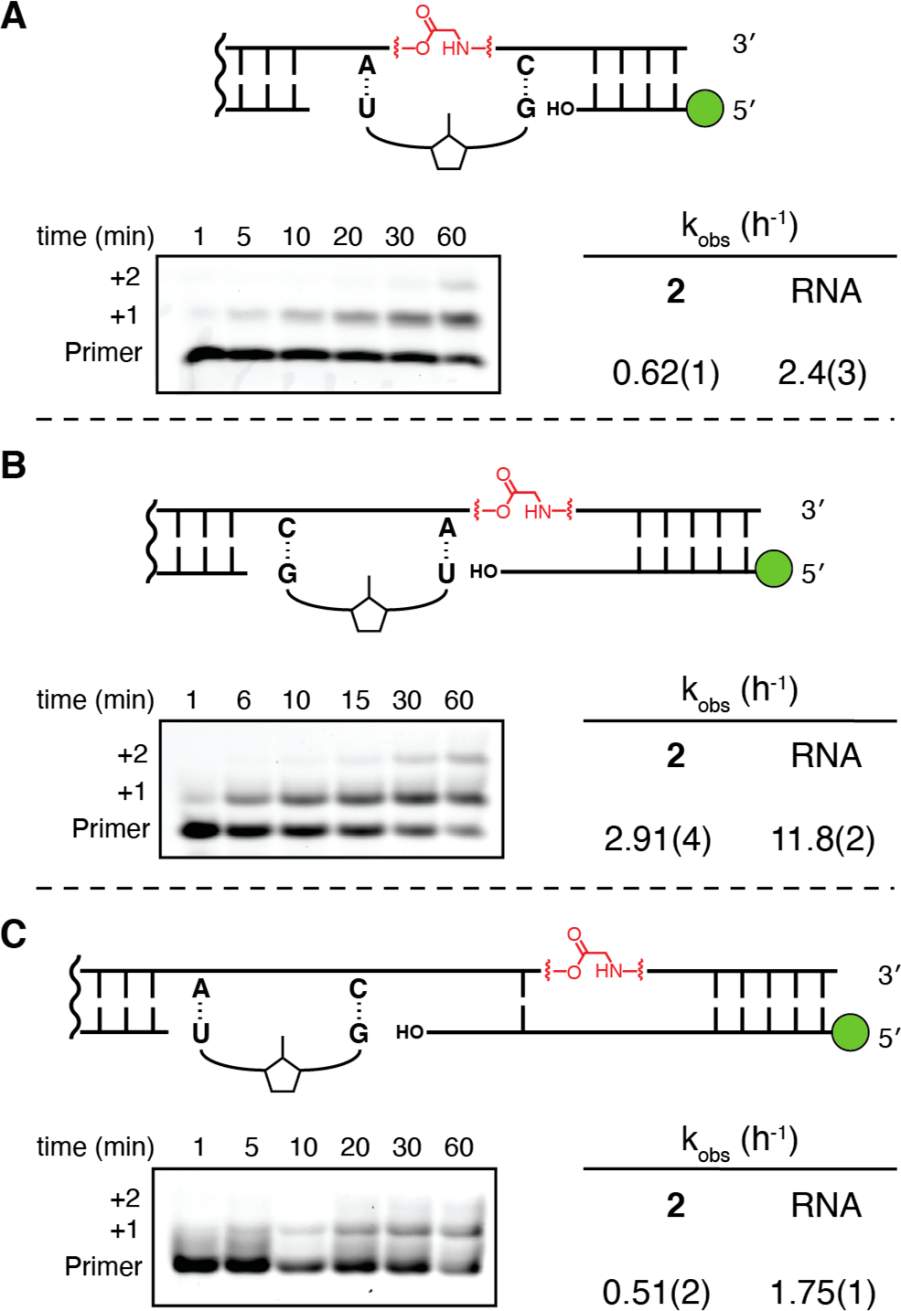
(A–C) Kinetic analysis of non-enzymatic primer extension across an RNA template containing a single glycine amino acid bridge. The top panel shows the schematic representation of the primer-template duplexes analyzed, showing the binding site for the G*U dimer in each case. RNA controls contained the identical primer, with the template identical except for the absence of the bridging amino acid. The bottom panel shows the time course of primer extension as monitored by polyacrylamide gel electrophoresis. **2** is the ssRNA template with a single glycine bridge. All reactions were performed at pH 8.0, 200 mM HEPES, and 100 mM MgCl_2_ with 20 mM G*U dimer. Values are reported as the mean with the standard deviation (*N* = 3) reported at the appropriate significant digit in parentheses. Gel images of RNA control reactions and plots used to determine kinetic parameters are shown in Figure S12.

To determine whether an RNA strand containing a single amino acid bridge could act as a template for RNA primer extension, we used the bridged 27mer ssRNA 2 (5′-AGAGAA-GAGAGCAGACA-gly-CCCGGCAGCU-3′), and an all-RNA control, as templates. The bridged template contains a stretch of residues 5′-ACA-gly-C-3′ such that only one activated dinucleotide intermediate, G*U, was necessary to compare template-directed copying at three positions. We tested three cases that differed only in the position of the primer 3′-end relative to the glycine bridge in the template. In the first case, the imidazolium-bridged G*U dinucleotide spans the glycine bridge (Figure 6A). In the second case, the glycine bridge is located after the primer annealing site (Figure 6B). In the third case, the primer extends over the glycine bridge (Figure 6C). Using 20 mM activated G*U dinucleotide and 100 mM Mg^2+^, we evaluated copying in all three cases. We observed primer extension in all three cases, implying that glycine-bridged RNA can indeed act as a template for RNA copying. We estimate that the kinetic defect due to copying over glycine-bridged RNA is approximately 4-fold relative to an all RNA control template for all three cases (Figure 6A–C and Figure S12).

## Discussion

We have found that the aminoacylation of an RNA primer can, under certain conditions, greatly enhance the non-enzymatic copying of an RNA template. The reaction of aminoacylated RNA primers with incoming imidazolium-bridged dinucleotides gives RNA products containing an amino acid “bridge” composed of a 5′ (C-terminal) ester linkage and a 3′ (N-terminal) phosphoramidate linkage. Notably, this copying reaction proceeds in the absence of Mg^2+^, which is damaging to protocell membranes at low millimolar concentrations. We have also examined the non-enzymatic, template-directed ligation of an aminoacylated RNA strand to a 2-methylimidizaole-activated ligator RNA. In this case, rates of ligation are enhanced by at least 2 orders of magnitude. Similar rate enhancements are seen with primers terminating in 3′-amino-2′,3′-dideoxyribonucleotides;^16^ however, no prebiotic synthesis of 3′-amino nucleotides has been described. In contrast, the aminoacylation of RNA is central to biology.

We employed Flexizyme-catalyzed acylation to obtain high yields of aminoacylated RNA primers for our studies. It is possible that RNA aminoacylation began to play a significant role in the RNA World only after the evolution of ribozymes became widespread, but an earlier process would open up the possibility of a role for aminoacylation chemistry in non-enzymatic RNA replication or ligation-mediated ribozyme assembly. The aminoacylation of RNA has been reported from mixtures of phosphorimidazolide-activated nucleotides, imidazole, and amino acids,^2^ but these reactions are quite inefficient. The discovery of more effective, prebiotically plausible chemistry for RNA aminoacylation would suggest the potential for a common role for this chemistry in the origins of both replication and translation. An efficient chemical aminoacylation process would also be experimentally useful if it overcame the sequence limitations enforced by our use of the Flexizyme ribozyme, which acylates only RNA terminating in CA-3′.

The regioselectivity of the phosphoramidate-forming primer extension and ligation reactions remains unknown. Our gel-based analysis cannot distinguish between reactions initiated from the 3′ and 2′ esters, because of the rapid transacylation of the initially formed aminoacyl ester. The different regioisomers, if a mixture indeed results from phosphoramidate formation, may display different templating activities and stabilities that have been conflated in this study.

It has been noted previously that the enhanced lifetime of the linkage of the aminoacyl ester to RNA upon formation of a neighboring phosphoramidate linkage may provide a mechanism for the stable integration of amino acid functionality into RNA.^9^ Our stability studies revealed the protective effect of duplex formation, which enhances the kinetic stability of amino acid-bridged RNA approximately 2-fold. Notably, high concentrations of Mg^2+^ promote degradation of the amino acid “bridge”; taken together with the much enhanced rates of RNA copying observed at low concentrations of Mg^2+^, this result suggests that amino acid-bridged RNA would accumulate preferentially under low-free Mg^2+^ conditions, conditions that are also most favorable for protocell stability. The major products of degradation of an amino acid “bridge” under RNA copying conditions are a 5′-fragment composed of native RNA, resulting from aminoacyl ester cleavage, and a 3′-fragment bearing an amino acid at the 5′-terminus linked by a phosphoramidate linkage. Such 5′-N-linked amino acids have been shown to be highly competent for further extension into peptides under activating conditions.^31^ Thus, phosphoramidate bond formation via either non-enzymatic primer extension or ligation, followed by hydrolysis of the aminoacyl ester, could initiate peptide synthesis, in addition to the functions outlined above.

We have shown that RNA containing an amino acid bridge remains competent as a template for further cycles of copying. It remains unknown whether amino acid-bridged RNA may serve a catalytic function. Ribozyme function can be enhanced using free amino acids as cofactors.^32^ In addition, introducing novel functional groups to RNA via chemical modification has proven to be a powerful approach for obtaining ribozymes with enhanced^33^ or new-to-nature functions.^34^ Our results show that non-enzymatic ligation with different amino acids can furnish RNA strands with bridging amino acids with a range of side chains. This novel route to the integration of amino acids within RNA may provide new opportunities for ribozyme catalysis that would be exciting to test.

## Supporting Information Available

Materials and Methods, Figures S1–S12, Table S1, and characterization of the G*U dinucleotide and 3,5-dinitrobenzyl esters of amino acids (PDF)
