# Supplementary material for: A Potential Role for Aminoacylation in Primordial RNA Copying Chemistry
Source: Biochemistry. 2021 Feb 1;60(6):477–88. doi: 10.1021/acs.biochem.0c00943 (PMC9634692; doi:10.1021/acs.biochem.0c00943)
Supplement: Supplementary file 1 — bi0c00943_si_001.pdf [file bi0c00943_si_001.pdf]

Supporting information

A Potential Role for Aminoacylation in Primordial RNA Copying Chemistry

Aleksandar Radakovic‡, Tom H. Wright‡, Victor S. Lelyveld and Jack W. Szostak*

Howard Hughes Medical Institute, Department of Molecular Biology and Center for Computational and Integrative Biology, Massachusetts General Hospital, Boston, Massachusetts 02114, United States; Department of Genetics, Harvard Medical School, Boston, Massachusetts 02115, United States

‡ These authors contributed equally
*To whom correspondence should be addressed:
CCIB 7215, Simches Research Center
185 Cambridge Street
Massachusetts General Hospital
Boston, Massachusetts 02114
E-mail: szostak@molbio.mgh.harvard.edu

Table of contents

Supplementary Figures S3

Supplementary Tables S22

Characterization of G*U dinucleotide and 3,5-dinitrobenzyl esters of amino acids S24

Supplementary Figures

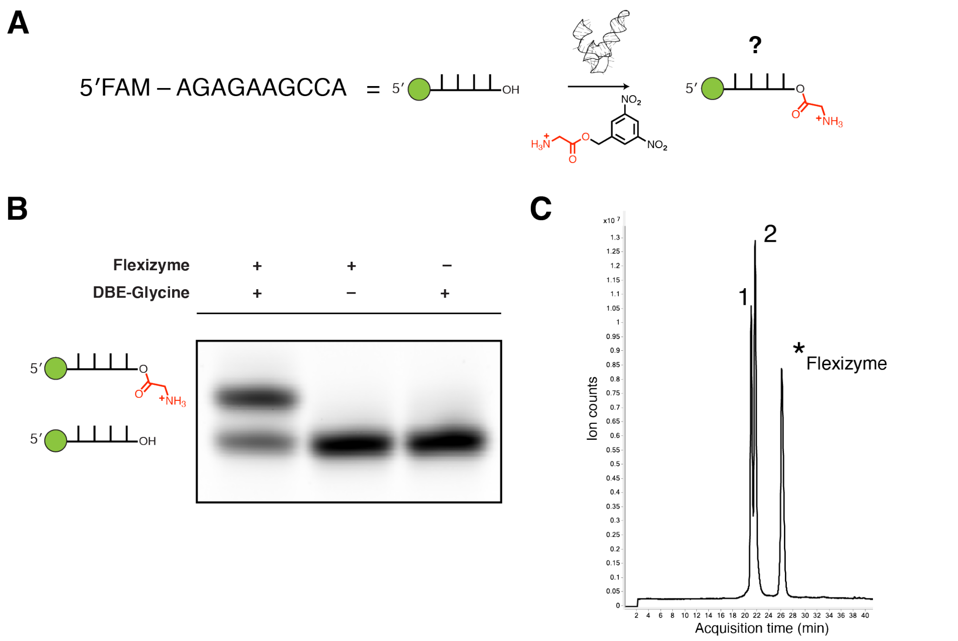

Primer	Calculated	Found	Signal
RNA	3765.65	3765.597	2
RNA – Gly	3822.67	3822.601	1
Flexizyme	14828.00	N/A	*

Figure S1
Flexizyme-catalyzed aminoacylation of RNA oligomers.

A. Schematic of the strategy used to aminoacylate RNA oligomers. Fluorescently-labeled RNA 10mer is subjected to aminoacylation by the dFx Flexizyme. Amino acid substrate of the dFx Flexizyme is the dinitrobenzyl ester of glycine (DBE-Gly).

B. Aminoacylation was monitored using 20% denaturing acid Urea-PAGE (0.1 M sodium acetate pH 5.0, 7 M Urea; running buffer 0.1 M sodium acetate pH 5.0). Under acidic conditions, the aminoacyl ester bond is sufficiently stable to enable gel resolution of the bands corresponding to the non-aminoacylated and aminoacylated RNA 10mer. Reaction conditions: 50 mM HEPES pH 8.0, 10 mM $MgCl_2$, 10 μM RNA 10mer, 5 mM DBE-Gly (20% final DMSO), 10 μM dFx, 14 h incubation on ice.

C. Aminoacylation was monitored using liquid chromatography coupled to a TOF mass spectrometer. Chromatographic resolution yielded 3 distinct signals in the total ion

chromatogram. The dominant molecular ions in signals labeled as 1 and 2 were observed to correspond to calculated exact masses of the aminoacylated RNA 10mer and the RNA 10mer, respectively. Signal * did not yield an interpretable m/z, likely due to it being out of the range of the mass detector and is presumed to be dFx.

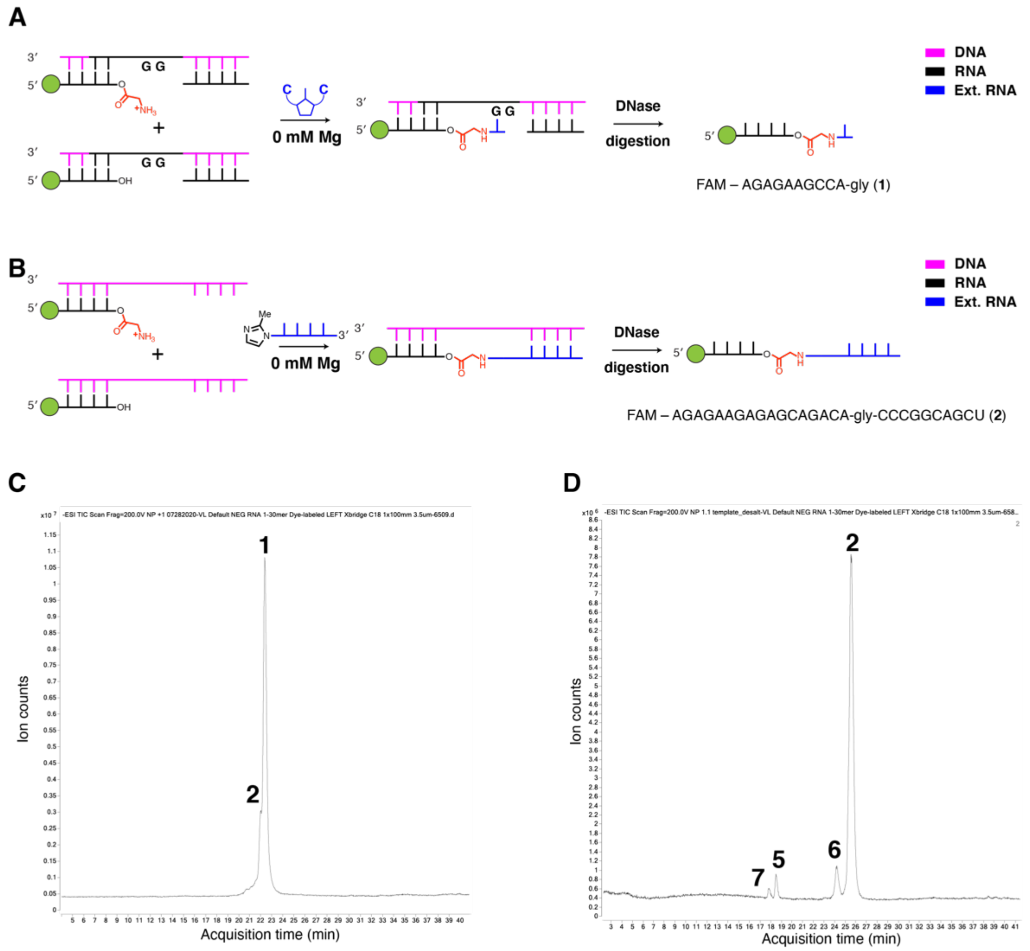

Oligo	Calculated	Found	Signal
**1**	4127.71	4127.6616	1
RNA	3765.65	3765.5988	2
RNA+1	4070.63	4070.6310	2

Oligo	Calculated	Found	Signal
**2**	9369.45	9369.2755	2
**6**	6117.00	6116.9553	6
**5**	3270.46	3270.4205	5
**7***	N/A	3373.4694	

*unknown species

Figure S2

Strategy to access glycine-linked +1 and +10 products.

A. Glycine-linked +1 product was accessed by performing a primer extension reaction (200 mM HEPES pH 8.0, with 13.5 mM C*C dimer; trace Mg^{2+} was chelated with a 20-fold excess EDTA to prevent the RNA reaction) on a mixture of aminoacylated and non-aminoacylated fluorescently-labeled RNA 10mer. The template was composed of DNA nucleotides, except for the 4 ribonucleotides in and adjacent to the templating region. TurboDNase was used to digest the DNA template and denaturing 20% Urea-PAGE was used to obtain product **1** with purity of 80-90%.

B. Glycine-linked +10 product was accessed by performing a ligation reaction (standard conditions except Mg^{2+} was chelated with a 20-fold excess EDTA to prevent the RNA reaction) on a mixture of aminoacylated and non-aminoacylated fluorescently-labeled RNA 17mer. The template was fully composed of DNA nucleotides. TurboDNase was used to digest the DNA template and denaturing 20% Urea-PAGE was used to obtain the product **2** with 90% purity.

C. Final product of the reaction shown in A was identified using liquid chromatography coupled to a TOF mass spectrometer. The total ion chromatogram showed two signals that were not fully chromatographically resolved, labeled 1 and 2. The molecular ion identified in signal 1 corresponded to the calculated exact mass of **1**, while the molecular ions in signal 2 corresponded to the calculated exact masses of the starting RNA 10mer and its +1 extension product. Purity of **1** was assessed based on normalized band intensities after gel electrophoresis (data not shown).

D. Final product of the reaction shown in B was identified using liquid chromatography coupled to a TOF mass spectrometer. The total ion chromatogram showed four signals that were fully chromatographically resolved, labeled 2, 6, 5, and 7 (labeling scheme corresponds to the one in Figure S4A). The molecular ion identified in signal 2 corresponded to the calculated exact mass of **2**. The observed molecular ions in signals 6, 5, and 7 and their identities are listed in the table below the chromatogram. Purity of **2** was assessed based on normalized band intensities after gel electrophoresis (data not shown).

A B

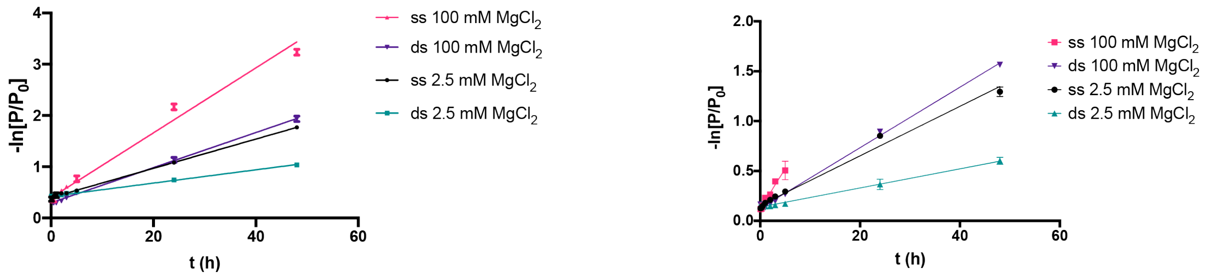

C

	Half life (h^{-1})	
	ssRNA	dsRNA
terminal N-P linkage (**1**)		
100 mM $MgCl_2$	10.9(3)	20.2(3)
2.5 mM $MgCl_2$	24.3(4)	53(1)
internal N-P linkage (**2**)		
100 mM $MgCl_2$	8.6(6)	22.8(2)
2.5 mM $MgCl_2$	20.1(9)	72(2)

Figure S3

Hydrolysis of glycine-linked +1 (**1**) and +10 (**2**) products at different $MgCl_2$ concentrations.

A. Hydrolytic stability of the terminal 2′(3′) glycyl phosphoramidate linkage in the glycine-linked +1 product. Shown is the plot of the negative natural logarithm of the ratio of oligomer **1** (P) to initial oligomer **1** (P_0) versus the time of reaction. The slope of the linear fits corresponded to k_{obs} for each of the tested conditions. Abbreviation ss = single-stranded; ds = double-stranded.

B. Hydrolytic stability of the internal 2′(3′) glycyl phosphoramidate linkage in the glycine-linked +10 product. Shown is the plot of the negative natural logarithm of the ratio of oligomer **2** (P) to initial oligomer **2** (P_0) versus the time of reaction. The slope of the linear fits corresponded to k_{obs} for each of the tested conditions. Abbreviation ss = single-stranded; ds = double-stranded. The single-stranded reaction at 100 mM $MgCl_2$ occurred rapidly enough to permit linear fitting and k_{obs} determination after 5 hours.

C. Summary of the obtained kinetic parameters from A and B. The half-lives were calculated from k_{obs} values by the following equation for a first-order process: $t_{1/2} = \ln(2)/k_{obs}$.

All reactions were performed at 200 mM HEPES pH 8.0.

A

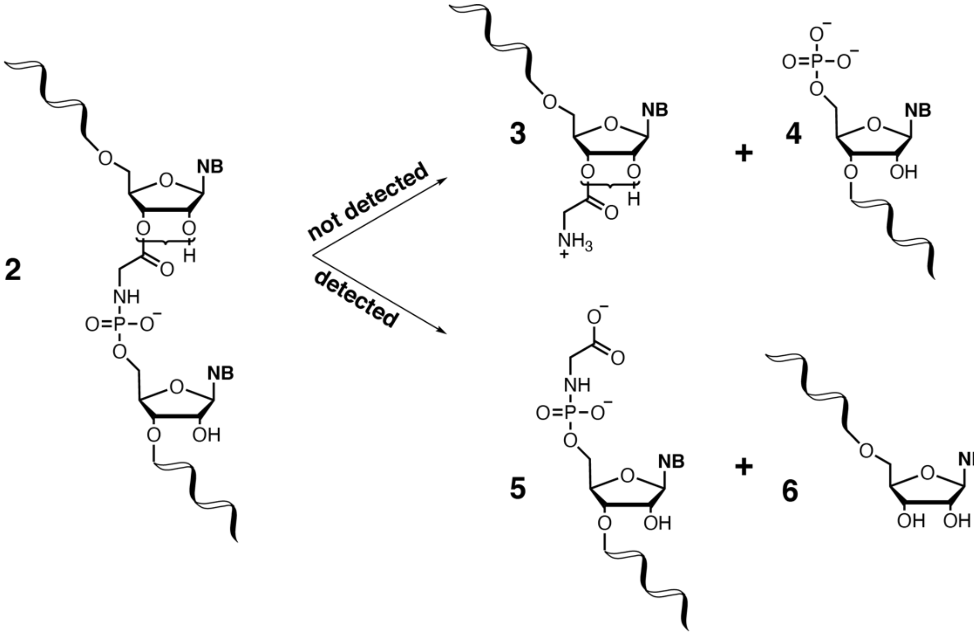

B

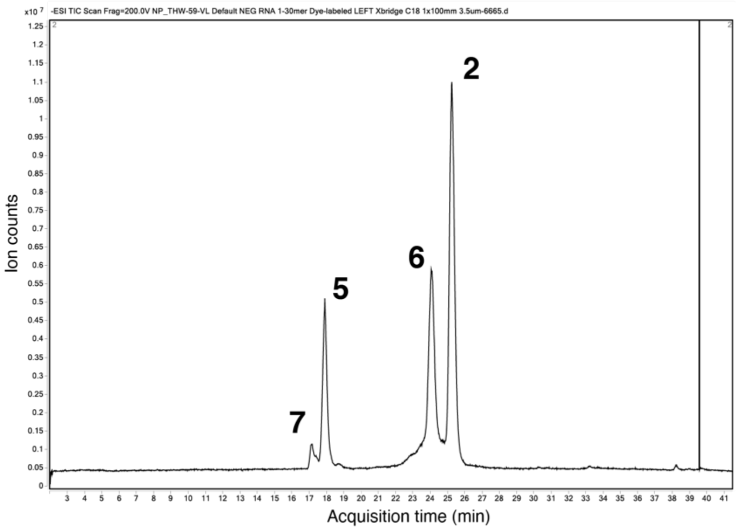

Compound	Calculated	Found
2	9369.45	9369.074
6	6117.00	6116.872
5	3270.46	3270.4055
7*	N/A	3373.4694

*unknown species

Figure S4

Preferential aminoacyl ester linkage hydrolysis of a glycine-linked RNA 27mer (**2**).

A. Potential degradation products of a glycine-linked RNA oligomer. Degradation can occur via the hydrolysis of the aminoacyl ester linkage or the phosphoramidate linkage to yield products that can be distinguished by liquid chromatography coupled to mass spectrometry.

B. Glycine-linked RNA 27mer (**2**) was incubated at pH 8.0, 200 mM HEPES, 2.5 mM $MgCl_2$ at 22 ºC for 24 h. The reaction was subjected to liquid chromatography coupled to a TOF mass spectrometer. The total ion chromatogram yielded four distinct, fully chromatographically resolved signals. The dominant molecular ions observed in each

signal and their identities based on calculated exact masses are listed in the shown table. The numbering in the chromatogram and the table follows the numbering from A. The observed signals corresponded to the expected products of the aminoacyl ester linkage hydrolysis. No signals that corresponded to the expected products of the phosphoramidate linkage hydrolysis were observed, suggesting that the phosphoramidate linkage is stable under typical primer extension and ligation reaction conditions.

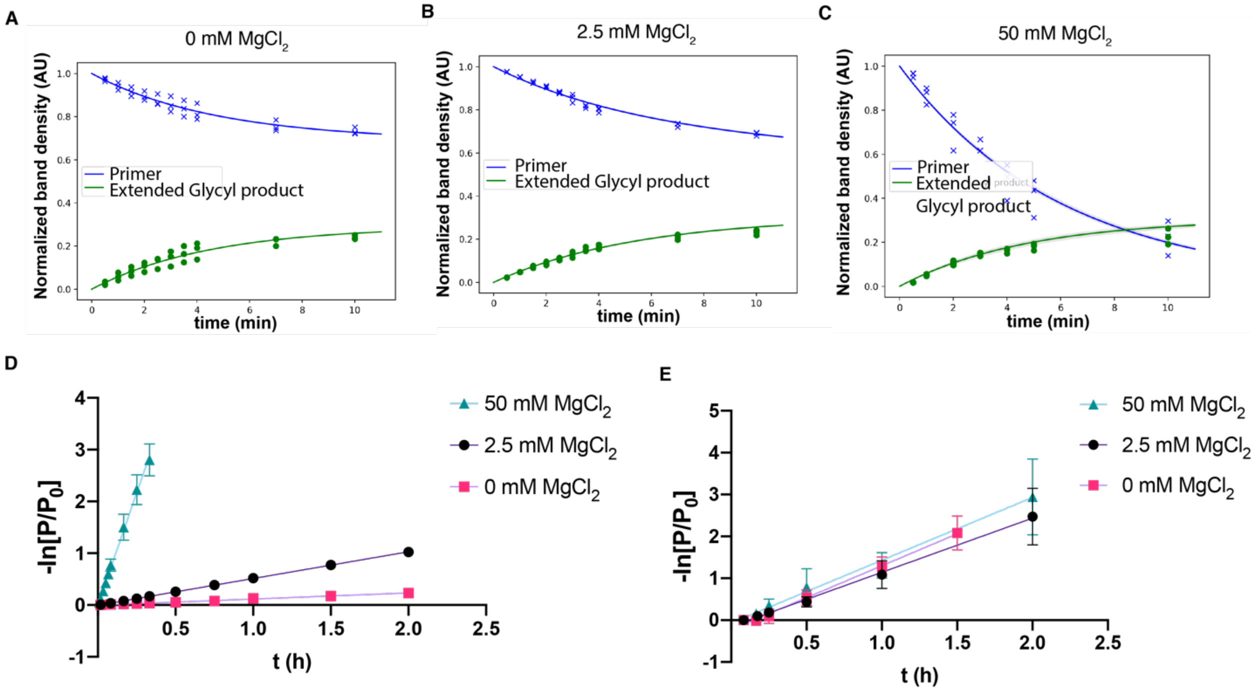

F

	h^{-1}		
Condition	k_1 (RNA-Gly)	k_3 (RNA)	k_2 (hydrolysis)
0 mM $MgCl_2$	12(1)	0.118(3)	1.5(3)
2.5 mM $MgCl_2$	9.8(3)	0.52(4)	1.3(3)
50 mM $MgCl_2$	11(1)	9(1)	1.3(2)

Figure S5

Kinetic analysis of nonenzymatic primer extension from 3′ hydroxyl and 2′(3′) glycyl terminated primers.

A.-C. Plots showing the normalized band intensities for the primer band, which includes both the primer-gly and primer species, (blue) and the extended glycyl +1 product (green) over the course of a primer extension reaction. Non-linear fits were obtained using the kinetic model described in the Materials and Methods section.

D. RNA primer control primer extension reaction. Shown is the plot of the negative natural logarithm of the ratio of primer (P) to initial primer (P_0) versus time of primer extension for a pure RNA primer at different $MgCl_2$ concentrations. The slope of the linear fits corresponded to k_{obs} for RNA reactions that we defined as k_3 in the kinetic model.

E. Primer-gly hydrolysis reaction. Shown is the plot of the negative natural logarithm of the ratio of primer-gly (P) to initial primer-gly (P_0) versus time of reaction for the glycine aminoacylated primer at different $MgCl_2$ concentrations. The slope of the linear fits corresponded to k_{obs} for primer-gly hydrolysis that we defined as k_2 in the kinetic model.
F. The tabulated summary of the obtained kinetic parameters from A-E plots. The values are reported as the mean +/− standard deviation from triplicate experiments.

The reactions in A-D were performed at 200 mM HEPES pH 8.0, with 20 mM C*C dimer. The hydrolysis reaction in E was performed at 200 mM HEPES pH 8.0, with the addition of 20 mM CMP instead of the C*C dinucleotide.

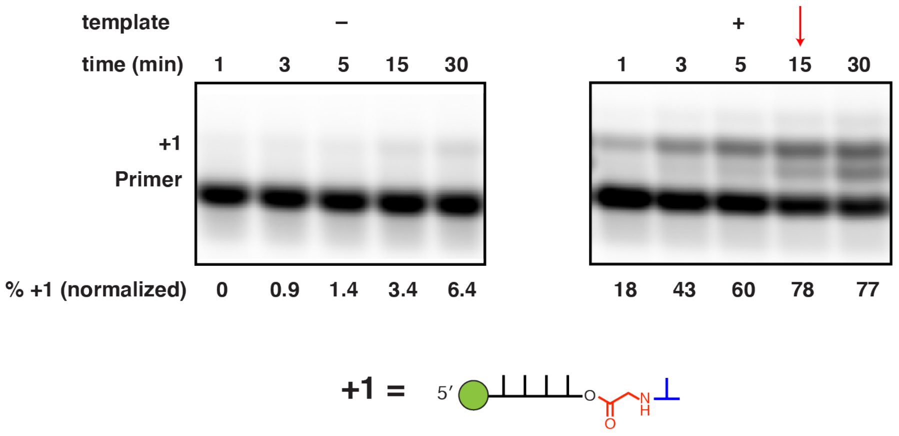

Figure S6

Off-template primer extension reaction of aminoacylated RNA 10mer primer.

A mixture of aminoacylated and non-aminoacylated fluorescently-labeled RNA 10mer primer was subjected to primer extension (200 mM HEPES pH 8.0, 20 mM C*C dimer, 2.5 mM $MgCl_2$) with or without adding the RNA template. Primer extension products were monitored by denaturing 20% Urea-PAGE. The red arrow indicates the point where the templated reaction reached completion. The percentage of the +1 product was normalized to acylation efficiency that was separately measured to be 34% using acidic Urea-PAGE.

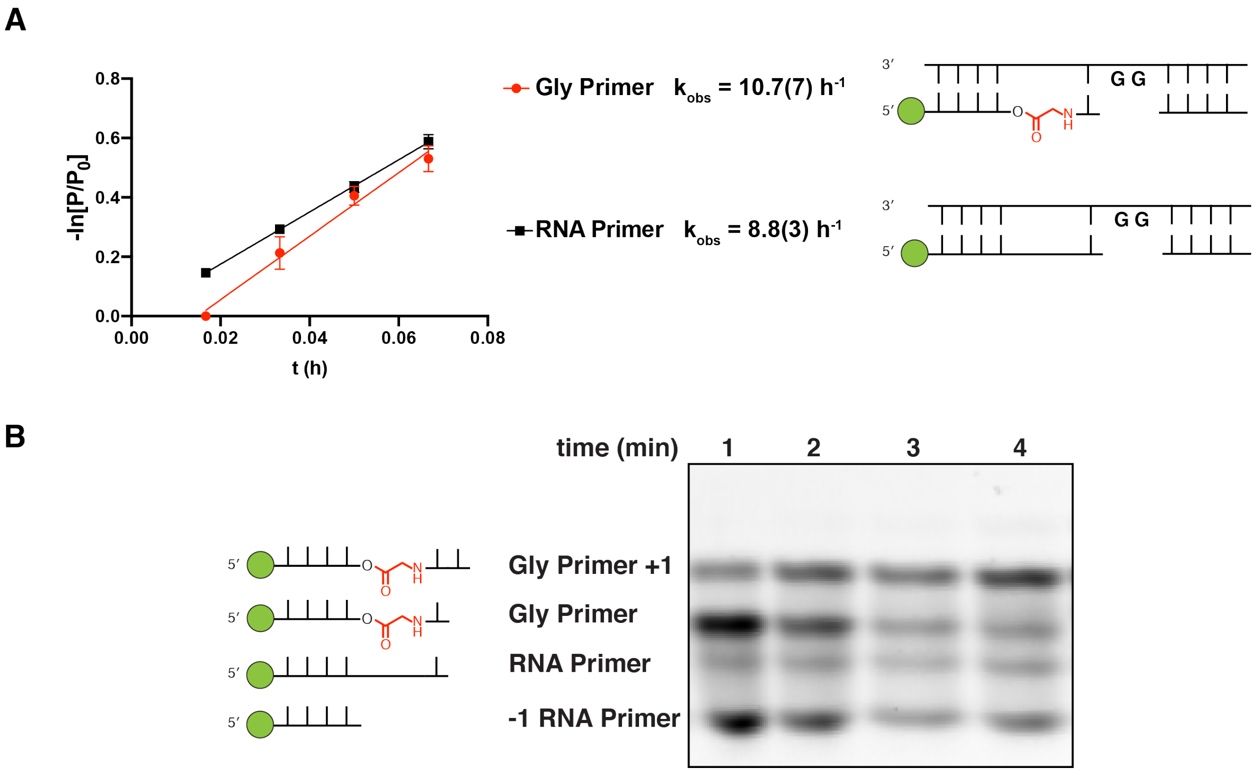

Figure S7

Kinetics of primer extension after the glycine linkage in the primer.

A. First-order kinetics of the RNA primer and glycine-linked primer extension reactions. Shown, on the left, is the plot of the negative natural logarithm of the ratio of primer (P) to initial primer (P_0) versus time of reaction. Gly primer reaction data are colored red. Pure RNA primer reaction data are colored black. The slope of the linear fits corresponded to k_{obs} for the two reactions. Shown, on the right, is the schematic representation of the Gly primer and the pure RNA primer aligned to the template before reaction initiation. All reactions were performed at 200 mM HEPES pH 8.0, 50 mM $MgCl_2$, with 20 mM C*C dimer. Values are reported as the mean +/– standard deviation from triplicate experiments.

B. Primer extension reactions were monitored by 20% denaturing Urea-PAGE. Shown gel is from the reaction of glycine-linked (Gly) primer (RNA reaction not shown). In addition to the Gly primer and its +1 extension product bands, the -1 RNA primer and its +1 extension product (RNA primer) bands were also observed. The -1 RNA primer is present due to the incomplete purification of the Gly primer (see Figures S2A and S2C for the synthesis and purification of Gly primer **1**). The data in A was obtained by monitoring the disappearance of the band labeled 'Gly Primer' (see **Kinetic analysis of primer extension reactions**).

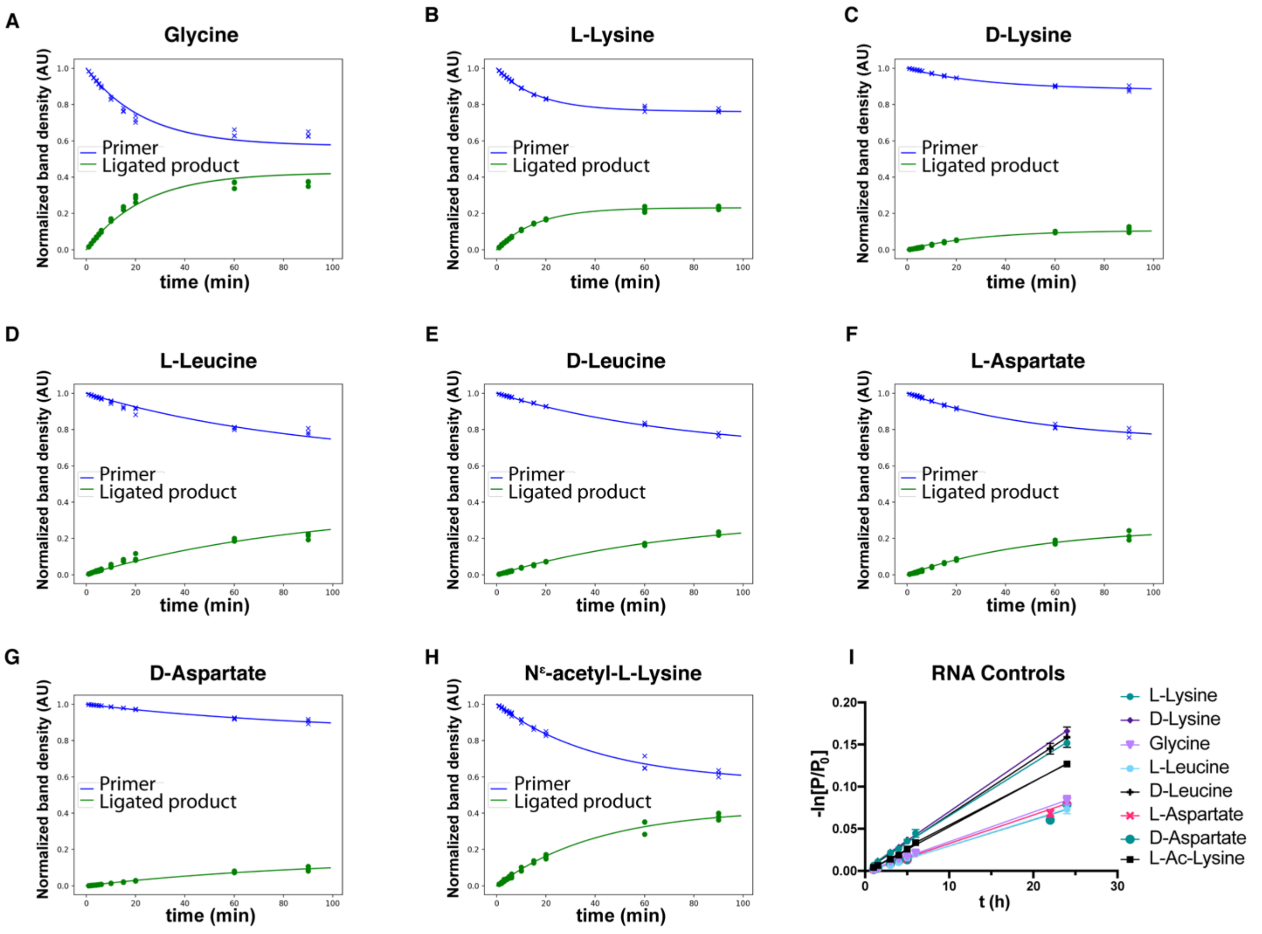

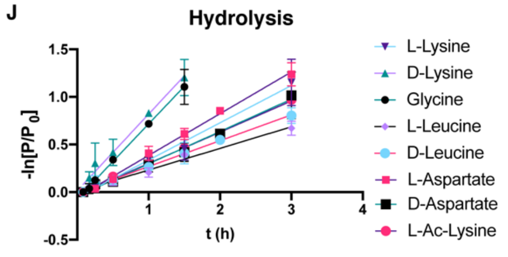

K

	h^{-1}		
Amino acid	k_1 (RNA-AA)	k_3 (RNA)	k_2 (hydrolysis)
Glycine	1.81(6)	0.0035(2)	0.79(8)
L-Lysine	3.37(8)	0.00625(6)	0.39(6)
D-Lysine	1.21(3)	0.0069(2)	0.80(6)
L-Leucine	0.455(5)	0.0029(2)	0.23(2)
D-Leucine	0.53(1)	0.0066(3)	0.27(3)
L-Aspartate	0.72(3)	0.0034(2)	0.44(3)
D-Aspartate	0.32(1)	0.0031(1)	0.34(5)
N^{ε}-acetyl-L-Lysine	1.12(2)	0.00536(5)	0.33(2)

Figure S8

Kinetic analysis of ligation from 3′ hydroxyl and 2′(3′) aminoacyl terminated primers.

A.-H. Plots showing the normalized band intensities for the primer band, which includes both the primer-amino acid and primer species, (blue) and the ligated product (green) over the course of a ligation reaction with 2-methylimidazole activated 10mer. Non-linear fits were obtained using the kinetic model described in the Materials and Methods section.

I. RNA primer control ligation reactions in the presence of dinitrobenzyl esters of each of the tested amino acids. Shown is the plot of the negative natural logarithm of the ratio of primer (P) to initial primer (P_0) versus time of ligation for a pure RNA primer. The slope of the linear fits corresponded to k_{obs} for RNA reactions that we defined as k_3 in the kinetic model.

J. Primer-amino acid hydrolysis reactions. Shown is the plot of the negative natural logarithm of the ratio of primer-amino acid (P) to initial primer-amino acid (P_0) versus time of reaction for the aminoacylated primer. The slope of the linear fits corresponded to k_{obs} for primer-amino acid hydrolysis that we defined as k_2 in the kinetic model.

K. The tabulated summary of the obtained kinetic parameters from A-I plots. The values are reported as the mean +/− standard deviation from triplicate experiments.

Reaction conditions: 2.5 mM $MgCl_2$, 200 mM HEPES pH 8.0, 2.5 μM primer, 3.75 μM template, 10 μM ligator. For the hydrolysis experiment 10 μM of unactivated ligator was used instead of the 2-metylimidazole activated ligator. Values are reported as the mean +/− standard deviation from triplicate experiments.

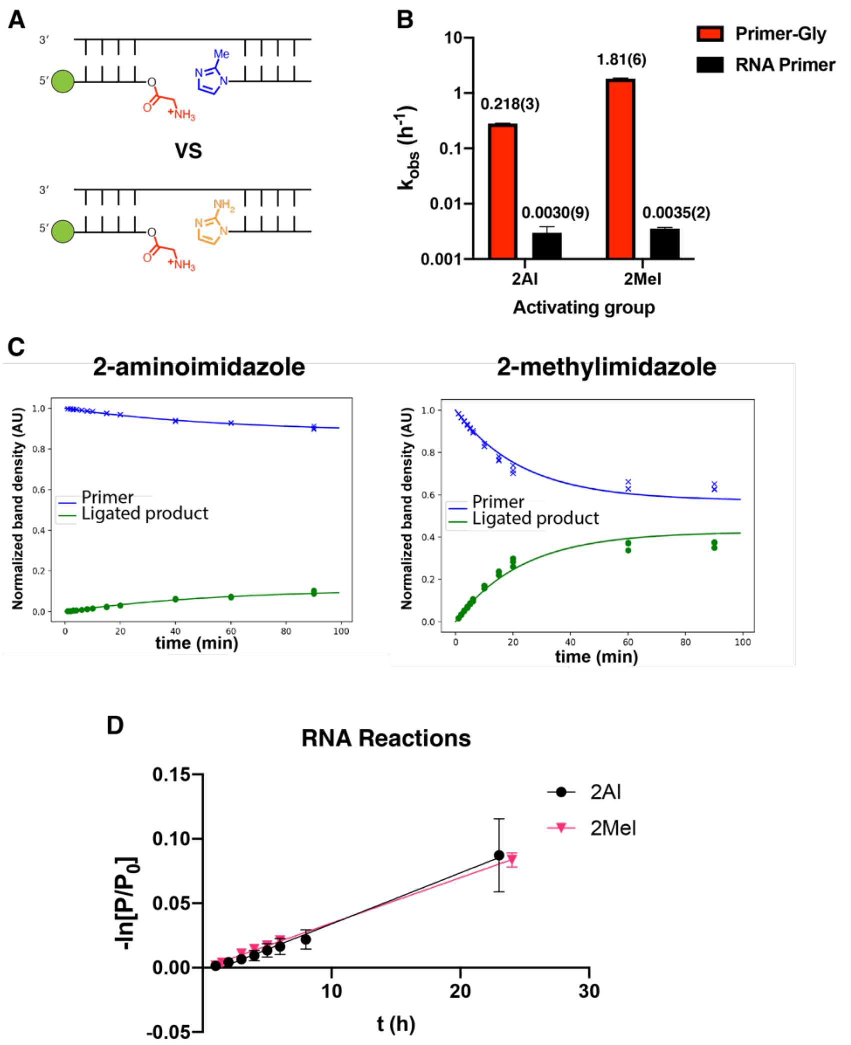

Figure S9

Kinetics of ligation reactions using 2-aminoimidazole or 2-methylimidazole activated 10mers.

A. Schematic of the reactions that were compared. Aminoacylated fluorescently-labeled 12mer RNA primers are shown. Pure non-aminoacylated 12mer RNA reactions were compared as well but are not shown in the diagram.

B. Observed rate constants for the ligation reactions of aminoacylated or non-aminoacylated 12mer RNA with either 2-aminoimidazole or 2-methylimidazole activated 10mers. Reaction conditions: 2.5 mM $MgCl_2$, 200 mM HEPES pH 8.0, 2.5 μM primer, 3.75 μM template, 10 μM activated ligator. Values are reported as the mean +/− standard deviation from triplicate experiments.

C. Plots showing the normalized band intensities for the primer band, which includes both the primer-gly and primer species, (blue) and the ligated product (green) over the course of a ligation reaction with 2-aminoimidazole (left) and 2-methylimidazole (right) activated oligonucleotides. Fits are obtained using the kinetic model described in the Materials and Methods section.

D. Pure RNA ligation reaction. Plot of the negative natural logarithm of the ratio of primer (P) to initial primer (P_0) versus time of ligation for a pure RNA primer with 2-aminoimidazole (black) and 2-methylimidazole (pink) activated oligonucleotides. The slope of the linear fits corresponds to k_{obs} for RNA.

Hydrolysis data for primer-gly can be found in Figure S8.

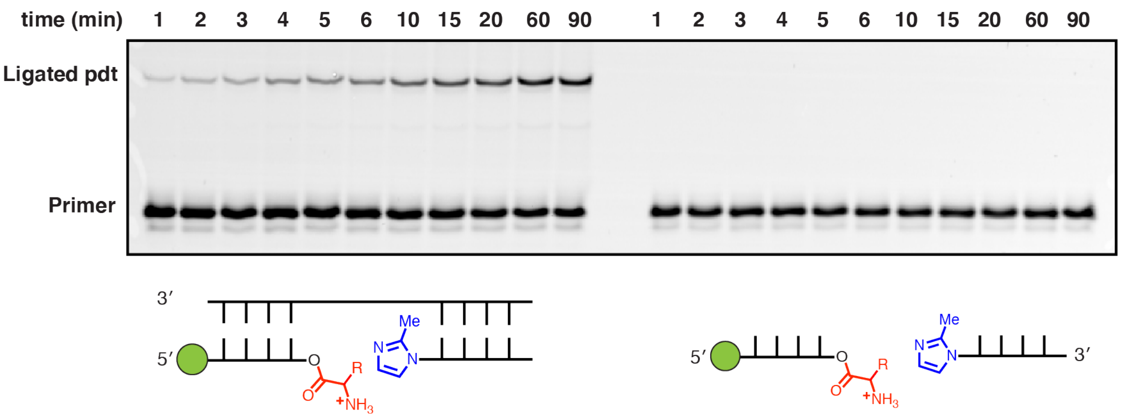

Figure S10

Off-template ligation reaction with aminoacylated fluorescently-labeled 12mer RNA primer and 2-methylimidazole activated 10mer. Reaction conditions were 2.5 mM $MgCl_2$, 200 mM HEPES pH 8.0, 2.5 μM primer, 10 μM activated ligator with (left) or without (right) 3.75 μM RNA template.

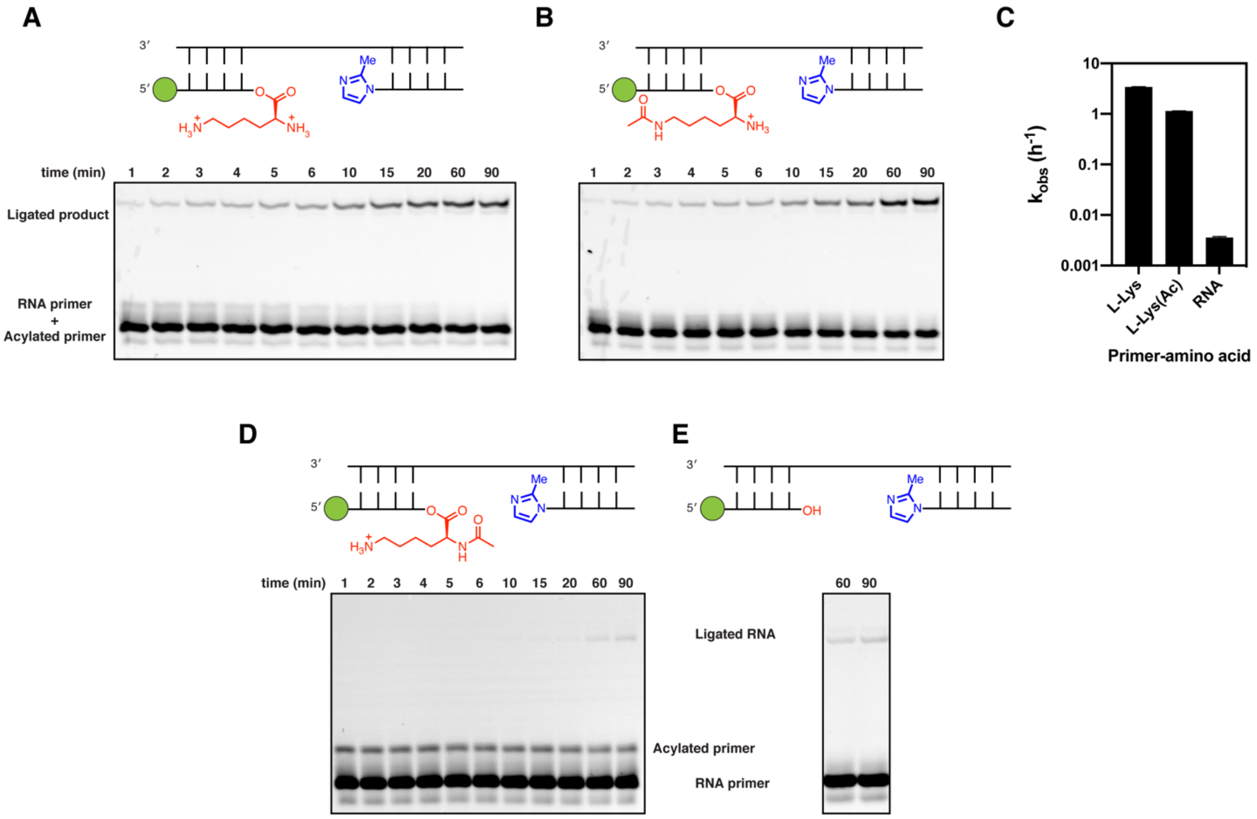

Figure S11

Ligation reactions with L-lysine and $N^{\varepsilon,\alpha}$-acetyl-L-lysine aminoacylated fluorescently-labeled 12mer RNA primer.

A.-B. A mixture of L-lysine or N^{ε}-acetyl-L-lysine aminoacylated and non-aminoacylated fluorescently-labeled 12mer RNA primer was subjected to ligation conditions and reaction progress was followed by 20% denaturing Urea-PAGE. The starting mixture of primers is indistinguishable by gel. The ligated product appears after 1 minute, consistent with the phosphoramidate bond formation.

C. A bar plot summarizing the rates of reactions shown in panels A and B using the kinetic model described in **Materials and Methods**. Reaction rates for hydrolysis and RNA controls for each of the two reactions can be found in Figure S8.

D. A mixture of N^{α}-acetyl-L-lysine aminoacylated and non-aminoacylated fluorescently-labeled 12mer RNA primer was subjected to ligation conditions and reaction progress was followed by 20% denaturing Urea-PAGE. The starting mixture of primers is

distinguishable by gel, presumably due to the greater stability of $N\alpha$-acetyl-L-lysine compared to L-lysine aminoacyl ester. Ligated product only appears after 60 minutes and it likely corresponds to the background RNA ligation shown in C.

E. A pure RNA 12mer primer was subjected to typical ligation conditions and reaction progress was followed by 20% denaturing Urea-PAGE. Ligated product is only visible after 60 minutes.

Reaction conditions: 2.5 mM $MgCl_2$, 200 mM HEPES pH 8.0, 2.5 μM primer, 3.75 μM template, 10 μM activated ligator.

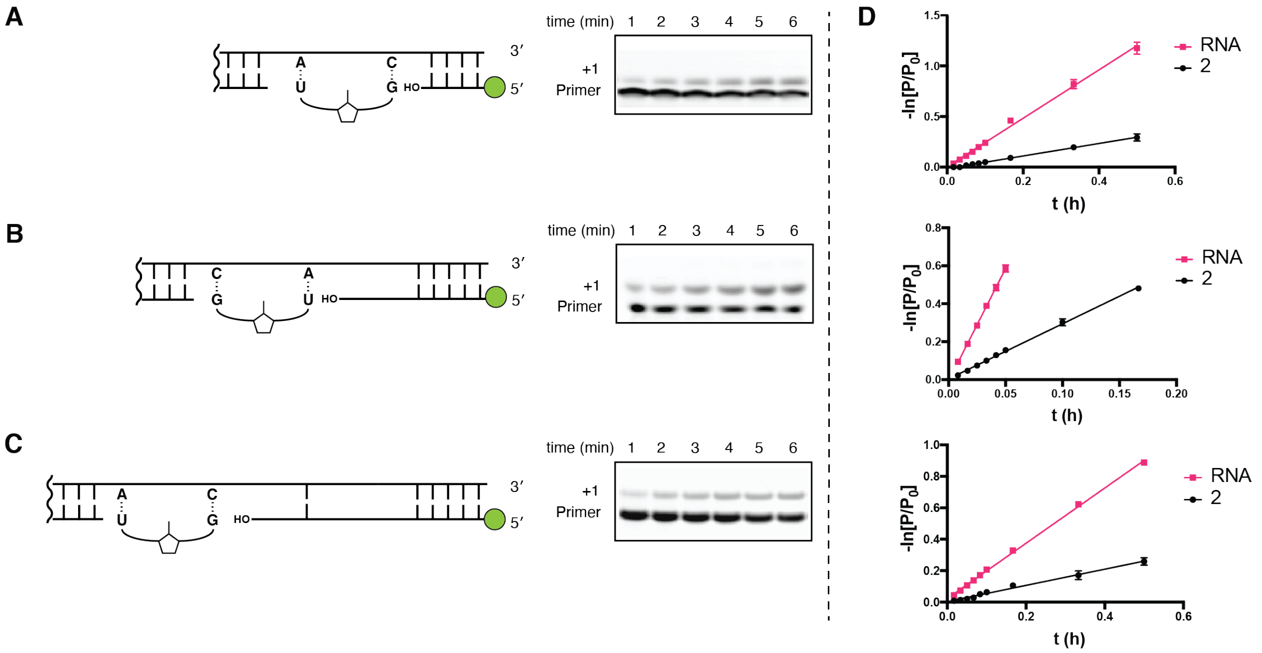

E

Scenario	k_{obs} (h-1) template **2**	k_{obs} (h-1) RNA template
A	0.62(1)	2.4(3)
B	2.91(4)	11.8(2)
C	0.51(2)	1.75(1)

Figure S12

A.-C. Kinetic analysis of nonenzymatic primer extension across an RNA template without the amino acid bridge. Left: Schematic representation of the primer-template duplexes analyzed, showing the binding site for the G*U dimer in each case. Right: The time course of primer extension was monitored using polyacrylamide gel electrophoresis. All reactions were performed at 200 mM HEPES pH 8.0, 100 mM $MgCl_2$, with 20 mM G*U dinucleotide.

D. First-order kinetic plots of primer extension reactions across a template containing a glycine bridge **2** (black) or RNA template without the bridge (pink). k_{obs} for each reaction was obtained from the slope of the linear fit of ln-transformed percentages of remaining primer over the course of reaction. Plots are aligned to correspond to the reactions in A-C.

E. Summary of obtained kinetic parameters from D. Values are reported as the mean +/– standard deviation from triplicate experiments.

Supplementary Tables

Table S1: Sequences used in this work. Nucleotides in magenta are deoxyribonucleotides, while all others are ribonucleotides.

**Name**	**Sequence**	**Use**
dFx Flexizyme	5′-GGAUCGAAAGAUUUCCGCAUCCCC GAAAGGGUACAUGGCGUUAGGU	Figure 2, 3, 4, S1, S2A
dFx Flexizyme M2	5′-GGAUCGAAAGAUUUCCGCAUCCCC GAAAGGGUACAUGGCGUUAGUU	Figures 5, 6, S2B, S8, S9, S10, S11
FX3 primer	5′FAM-AGAGAAGCCA	Figures 2, 3, 4, S1, S2A, S5, S6
FX_T2 template	5′-UAAUCCAAGGUGGCUUCUCU	Figures 2, 3, 4
FX_S2 sandwich	5′-UUGGAUUA	Figures 2, 3, 4
LXP1F primer	5′FAM-AGAGAGCAGACA	Figures 5, S8, S9, S10, S11
NP Lig T template	5′-AGCUGCCGGGUGUCUGCUCUCU	Figures 5, S3, S8, S9, S10, S11; Table 1 (ds **2**)
Ligator1	5′-2MeImid (or 2AminoImid)CCCGGCAGCU	Figures 5, S2B, S8, S9, S10, S11
LXP2F primer	5′FAM-AGAGAAGAGAGCAGACA	Figures 6, S2B
NP DNA T template	5′-AGCTGCCGGGTGTCTGCTCTCT	Figure S2B
FX3+1 primer	5′FAM-AGAGAAGCCAC	Figure S7
FX3+gly1 (**1**) primer	5′FAM-AGAGAAGCCAglyC	Figures S2A, S3, S7; Table 1
FX_T2 hybrid template	5′-TAATCCAAGGUGGCTTCTCT	Figure S2A
FX_T5 template	5′-UAAUCCAGGGUGGCUUCUCU	Figure S7; Table 1 (ds **1**)

FX_S5 sandwich	5′-UGGAUUA	Figure S7
NP FX1 primer	5′FAM-AGCUGCCGG	Figure 6A, S12A
NP FX1 sandwich	5′-GUCUGCUCUCU	Figure 6A, S12A
NP FX2 primer	5′FAM-AGCUGCCGGG	Figure 6B, S12B
NP FX2 sandwich	5′-UCUGCUCUCU	Figure 6B, S12B
NP FX3 primer	5′FAM-AGCUGCCGGGU	Figure 6C, S12C
NP FX3 sandwich	5′-CUGCUCUCU	Figure 6C, S12C
NP RNA T template	5′-AGAGAGCAGACACCCGGCAGCU	RNA control template for Figure 6
NP template (**2**)	5′-AGAGAGCAGACAglyCCCGGCAGCU	Figure 6 template, S3, S4, S12; Table 1

Characterization of G*U dinucleotide and 3,5-dinitrobenzyl esters of amino acids

G*U 2-aminoimidazolium dinucleotide

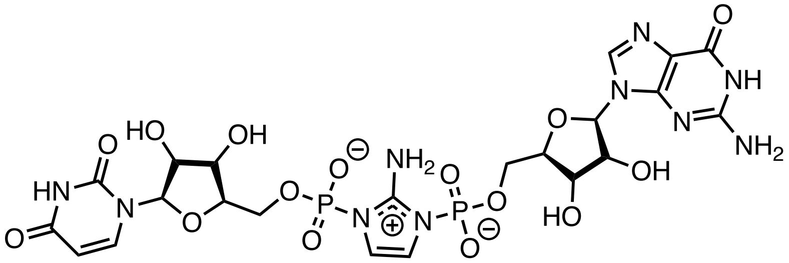

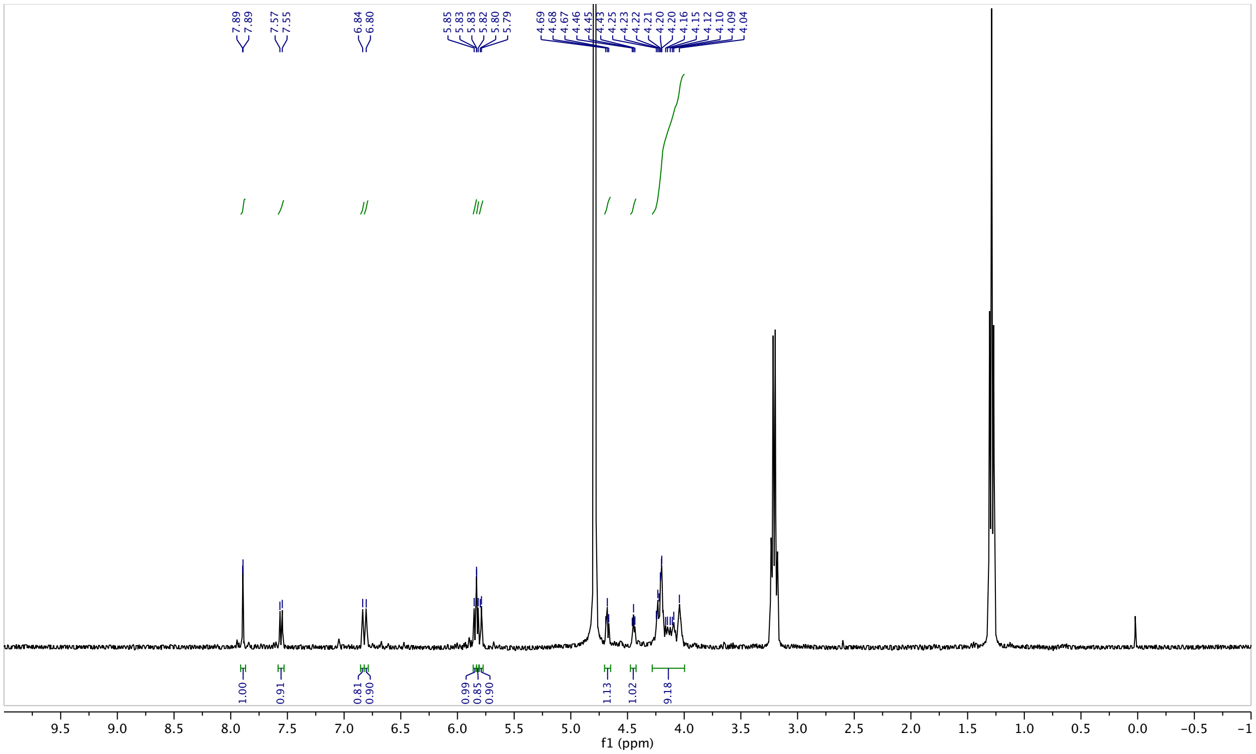

^{1}H NMR (400 MHz, D_2O) δ 7.89 (d, J = 1.3 Hz, 1H), 7.56 (d, J = 8.4 Hz, 1H), 6.84 (s, 1H), 6.80 (s, 1H), 5.84 (d, J = 8.3 Hz, 1H), 5.82 (d, J = 5.3 Hz, 1H), 5.79 (d, J = 4.3 Hz, 1H), 4.68 (t, J = 5.3 Hz, 1H), 4.45 (t, J = 4.7 Hz, 1H), 4.28 – 4.00 (m, 9H); peaks corresponding to residual TEAB were observed at 3.21 ppm and 1.29 ppm. LRMS (ESI- ion trap): calc for $[C_{22}H_{27}N_{10}O_{15}P_2]^-$ 733.11, found 733.2. HRMS (ESI-TOF): calc for $[C_{22}H_{27}N_{10}O_{15}P_2]^-$ 733.1138, found 733.1141.

G*U 2-aminoimidazolium dinucleotide

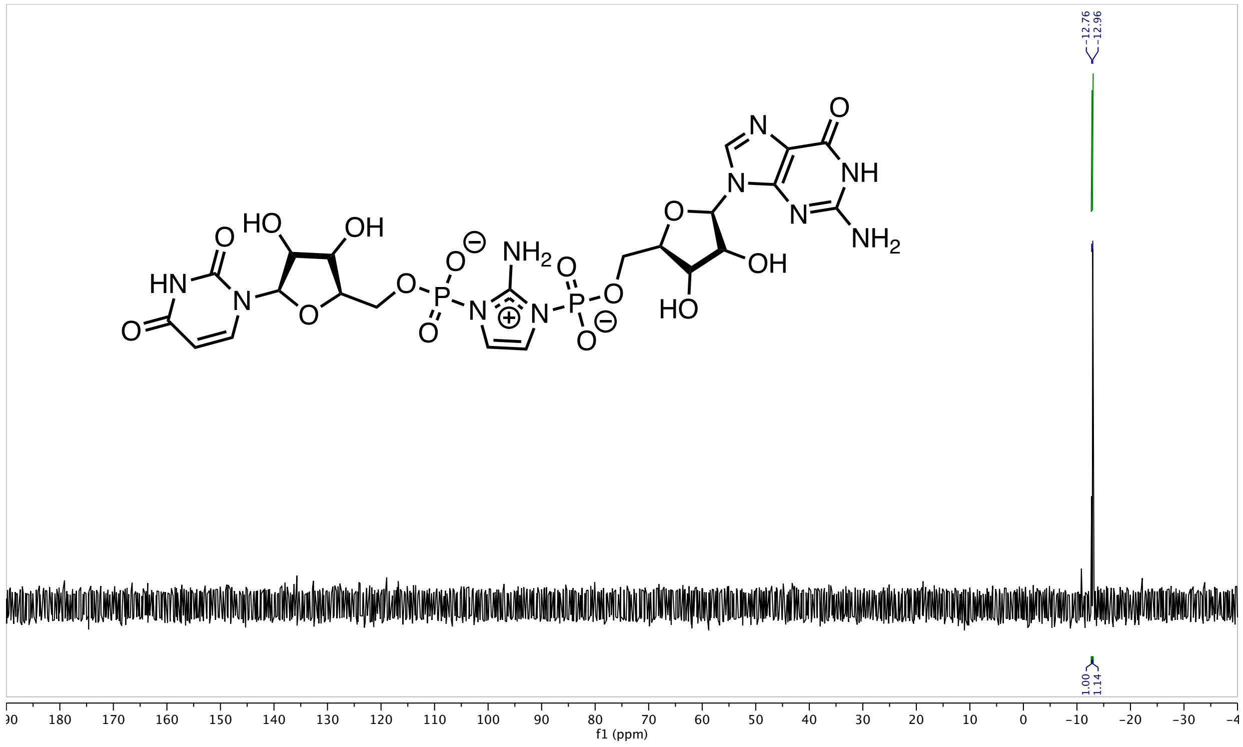

^{31}P NMR (162 MHz, D_2O) δ -12.76, -12.96.

(*S*)-3-amino-4-((3,5-dinitrobenzyl)oxy)-4-oxobutanoic acid or L-aspartate-DBE

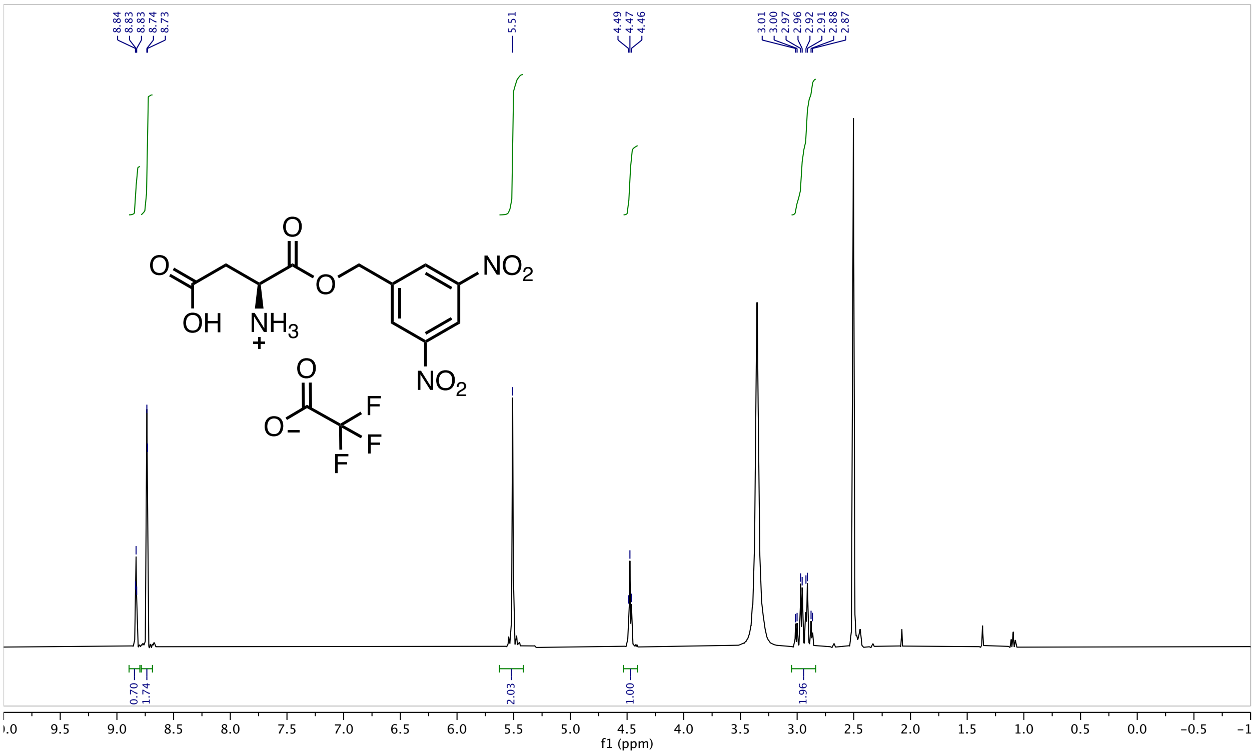

^{1}H NMR (400 MHz, dmso) δ 8.83 (t, *J* = 2.1 Hz, 1H), 8.73 (d, *J* = 2.1 Hz, 2H), 5.51 (s, 2H), 4.47 (t, *J* = 5.1 Hz, 1H), 2.94 (qd, *J* = 18.0, 5.1 Hz, 2H). LRMS (ESI- ion trap): calc for $[C_{11}H_{11}N_3O_8 + (H^+)]^+$ 314.06, found 314.0. HRMS (ESI-TOF): calc for $[C_{11}H_{11}N_3O_8 + (H^+)]^+$ 314.06244, found 314.0642.

(*R*)-3-amino-4-((3,5-dinitrobenzyl)oxy)-4-oxobutanoic acid or D-aspartate-DBE

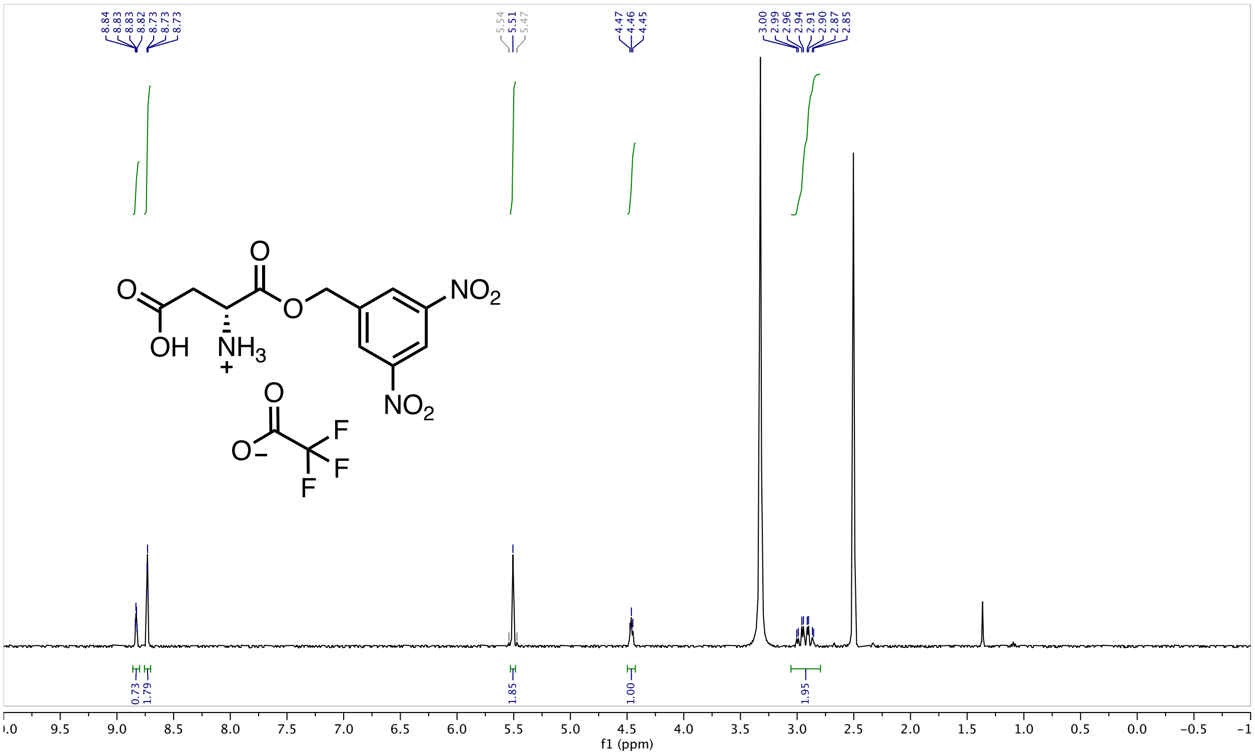

^{1}H NMR (400 MHz, dmso) δ 8.83 (q, J = 2.0 Hz, 1H), 8.73 (d, J = 1.9 Hz, 2H), 5.51 (s, 2H), 4.46 (t, J = 5.1 Hz, 1H), 2.93 (qd, J = 17.8, 4.9 Hz, 2H). LRMS (ESI- ion trap): calc for $[C_{11}H_{11}N_3O_8 + (H^+)]^+$ 314.06, found 314.0. HRMS (ESI-TOF): calc for $[C_{11}H_{11}N_3O_8 + (H^+)]^+$ 314.06244, found 314.0639.

3,5-dinitrobenzyl glycinate or glycine-DBE

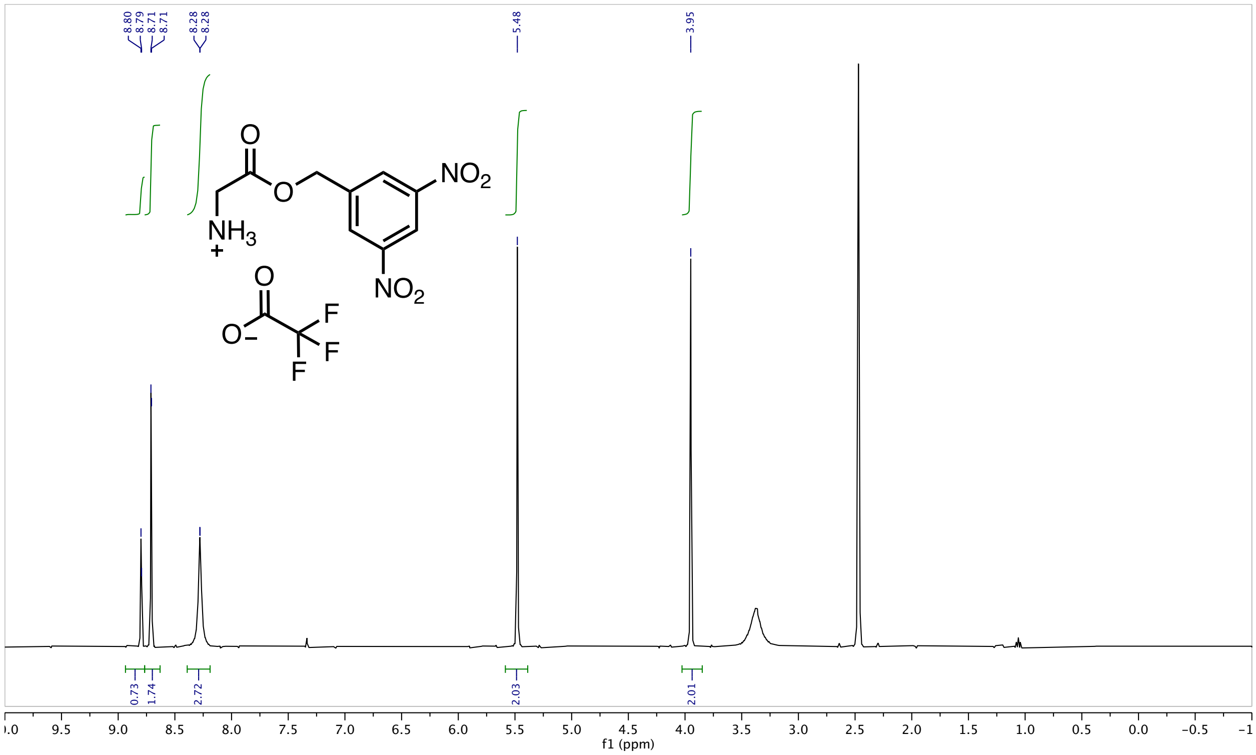

^{1}H NMR (400 MHz, dmso) δ 8.80 (d, J = 2.2 Hz, 1H), 8.71 (d, J = 2.1 Hz, 2H), 8.39 – 8.19 (m, 3H), 5.48 (s, 2H), 3.95 (s, 2H). LRMS (ESI- ion trap): calc for $[C_9H_9N_3O_6 + (H^+)]^+$ 256.06, found 255.9. HRMS (ESI-TOF): calc for $[C_9H_9N_3O_6 + (H^+)]^+$ 256.05696, found 256.0582.

3,5-dinitrobenzyl *L*-leucinate or L-leucine-DBE

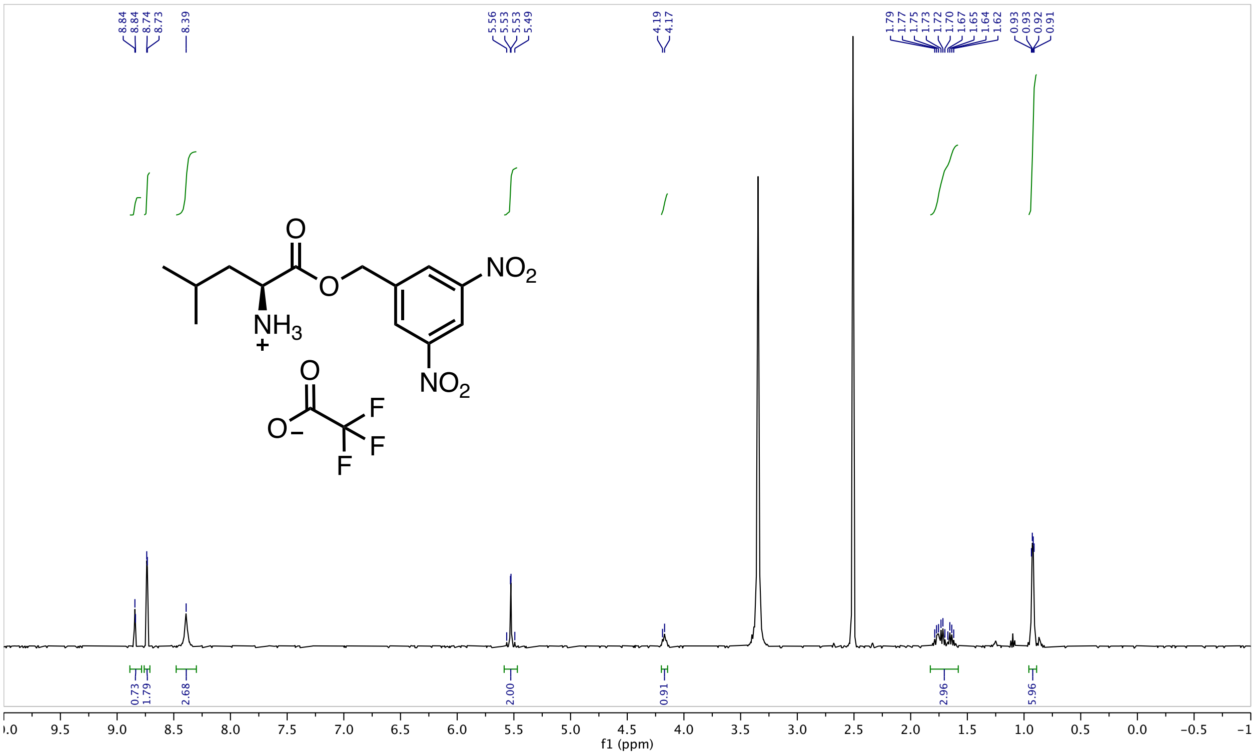

^{1}H NMR (400 MHz, dmso) δ 8.84 (d, *J* = 2.1 Hz, 1H), 8.74 (d, *J* = 2.1 Hz, 2H), 8.39 (s, 3H), 5.53 (d, *J* = 1.9 Hz, 2H), 4.18 (d, *J* = 7.2 Hz, 1H), 1.83 – 1.58 (m, 3H), 0.92 (dd, *J* = 6.3, 3.0 Hz, 6H). LRMS (ESI- ion trap): calc for $[C_{13}H_{17}N_3O_6 + (H^{+})]^{+}$ 312.12, found 312.1. HRMS (ESI-TOF): calc for $[C_{13}H_{17}N_3O_6 + (H^{+})]^{+}$ 312.11956, found 312.1218.

3,5-dinitrobenzyl *D*-leucinate or D-leucine-DBE

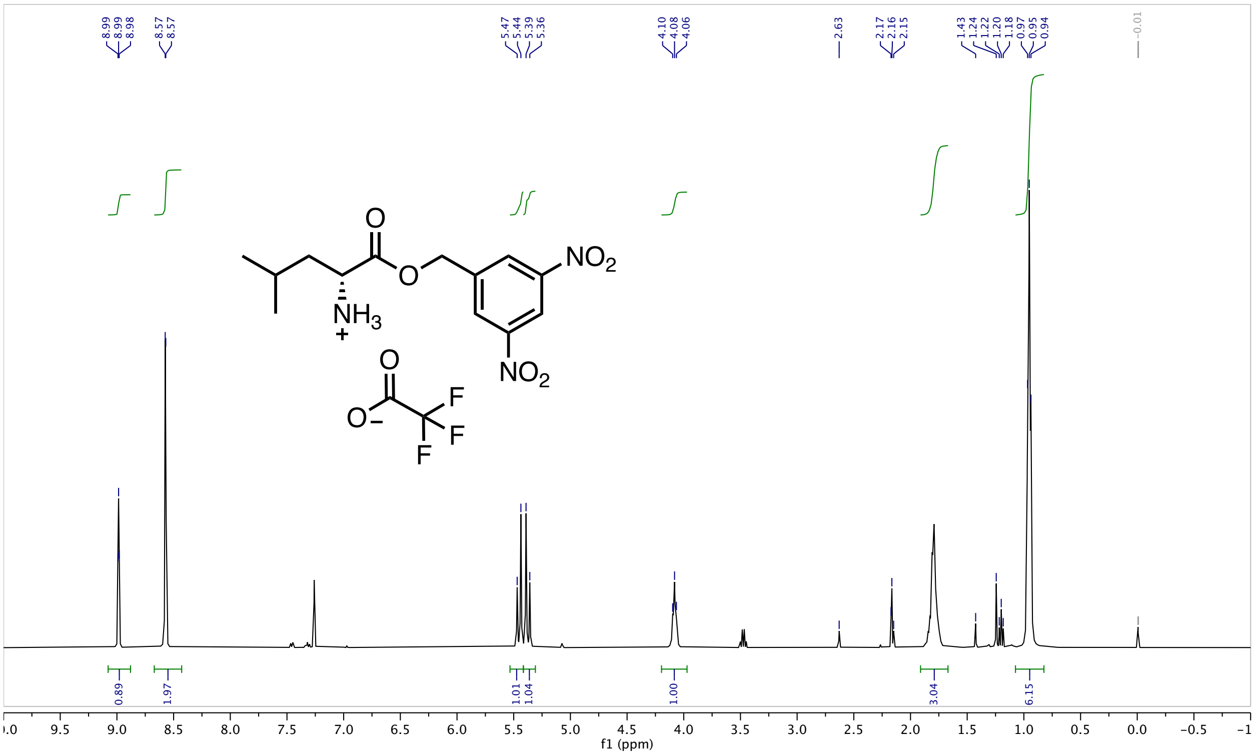

^{1}H NMR (400 MHz, cdcl$_3$) δ 8.99 (t, *J* = 2.1 Hz, 1H), 8.57 (d, *J* = 2.1 Hz, 2H), 5.45 (d, *J* = 13.2 Hz, 1H), 5.37 (d, *J* = 13.2 Hz, 1H), 4.20 – 3.97 (m, 1H), 1.79 (p, *J* = 7.7, 6.7 Hz, 3H), 0.95 (t, *J* = 6.0 Hz, 6H). LRMS (ESI- ion trap): calc for $[C_{13}H_{17}N_3O_6 + (H^+)]^+$ 312.12, found 312.1. HRMS (ESI-TOF): calc for $[C_{13}H_{17}N_3O_6 + (H^+)]^+$ 312.11956, found 312.1210.

3,5-dinitrobenzyl *L*-lysinate or L-lysine-DBE

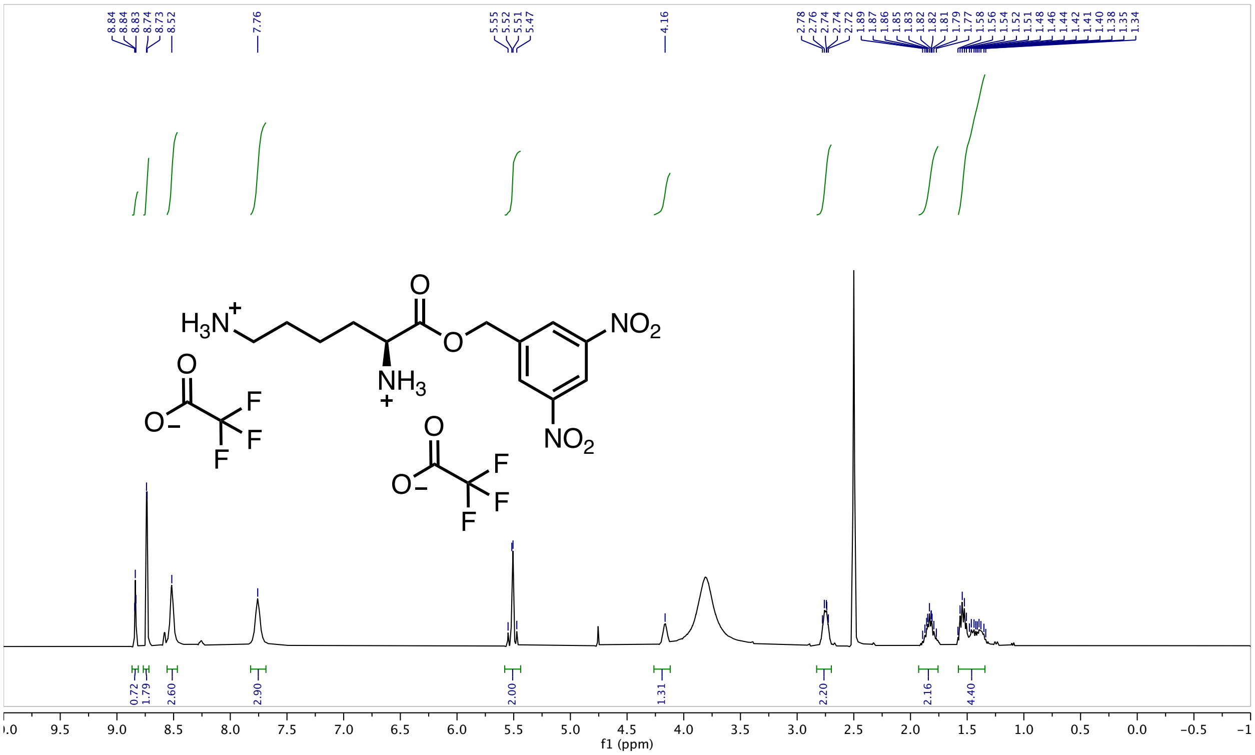

^{1}H NMR (400 MHz, dmso) δ 8.84 (t, *J* = 2.2 Hz, 1H), 8.74 (d, *J* = 2.1 Hz, 2H), 8.52 (s, 3H), 7.76 (s, 3H), 5.58 – 5.44 (m, 2H), 4.16 (s, 1H), 2.75 (q, *J* = 6.8 Hz, 2H), 1.83 (qd, *J* = 9.5, 6.1 Hz, 2H), 1.58 – 1.34 (m, 4H). LRMS (ESI- ion trap): calc for [$C_{13}H_{18}N_4O_6$ + (H^+)]$^+$ 327.13, found 327.1. HRMS (ESI-TOF): calc for [$C_{13}H_{18}N_4O_6$ + (H^+)]$^+$ 327.13046, found 327.1312.

3,5-dinitrobenzyl *D*-lysinate or D-lysine-DBE

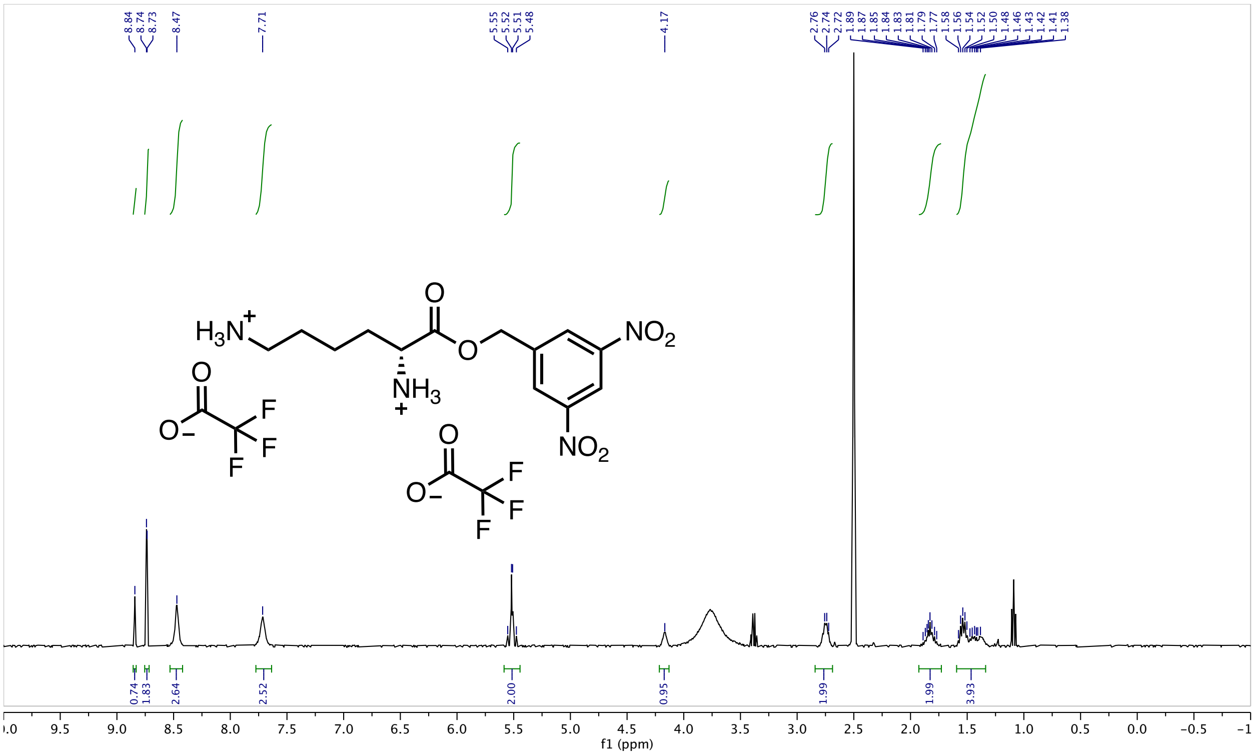

^{1}H NMR (400 MHz, dmso) δ 8.84 (s, 1H), 8.74 (d, *J* = 2.1 Hz, 2H), 8.47 (s, 3H), 7.71 (s, 3H), 5.59 – 5.44 (m, 2H), 4.17 (s, 1H), 2.84 – 2.69 (m, 2H), 1.83 (dh, *J* = 16.9, 7.8 Hz, 2H), 1.49 (ddt, *J* = 36.9, 20.7, 10.9 Hz, 4H). LRMS (ESI- ion trap): calc for $[C_{13}H_{18}N_4O_6 + (H^+)]^+$ 327.13, found 327.1. HRMS (ESI-TOF): calc for $[C_{13}H_{18}N_4O_6 + (H^+)]^+$ 327.13046, found 327.1329.

3,5-dinitrobenzyl acetyl-*L*-lysinate or L-acetyllysine-DBE

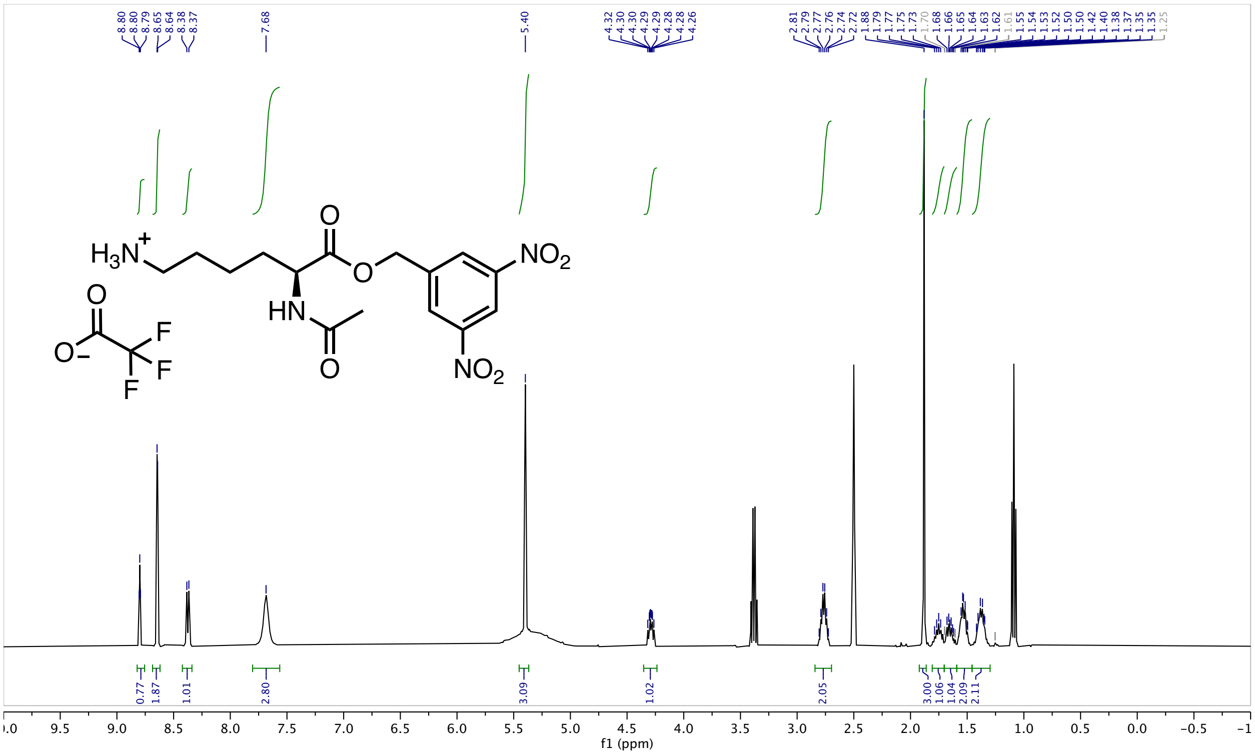

^{1}H NMR (400 MHz, dmso) δ 8.80 (t, J = 2.1 Hz, 1H), 8.64 (d, J = 2.1 Hz, 2H), 8.37 (d, J = 7.2 Hz, 1H), 7.68 (s, 3H), 5.40 (s, 3H), 4.29 (ddd, J = 9.0, 7.1, 5.2 Hz, 1H), 2.76 (h, J = 6.1 Hz, 2H), 1.88 (s, 3H), 1.76 (dq, J = 13.7, 6.7 Hz, 1H), 1.70 – 1.59 (m, 1H), 1.52 (td, J = 8.6, 3.7 Hz, 2H), 1.38 (q, J = 6.7, 5.4 Hz, 2H). LRMS (ESI- ion trap): calc for $[C_{15}H_{20}N_4O_7 + (H^+)]^+$ 369.14, found 369.1. HRMS (ESI-TOF): calc for $[C_{15}H_{20}N_4O_7 + (H^+)]^+$ 369.14102, found 369.1430.
